# An integrated model system to gain mechanistic insights into biofilm-associated antimicrobial resistance in *Pseudomonas aeruginosa* MPAO1

**DOI:** 10.1038/s41522-020-00154-8

**Published:** 2020-10-30

**Authors:** Adithi R. Varadarajan, Raymond N. Allan, Jules D. P. Valentin, Olga E. Castañeda Ocampo, Vincent Somerville, Franziska Pietsch, Matthias T. Buhmann, Jonathan West, Paul J. Skipp, Henny C. van der Mei, Qun Ren, Frank Schreiber, Jeremy S. Webb, Christian H. Ahrens

**Affiliations:** 1 Research Group Molecular Diagnostics Genomics & Bioinformatics, Agroscope and SIB Swiss Institute of Bioinformatics, Wädenswil, Switzerland; 2 School of Biological Sciences and Institute for Life Sciences, University of Southampton, Southampton, SO17 1BJ UK; 3 National Biofilms Innovation Centre, University of Southampton, Southampton, SO17 1BJ UK; 4 School of Pharmacy, Faculty of Health and Life Sciences, De Montfort University, Leicester, LE1 9BH UK; 5 Laboratory for Biointerfaces, Empa, Swiss Federal Laboratories for Materials Science and Technology, St. Gallen, Switzerland; 6 Department of BioMedical Engineering, University of Groningen and University Medical Center Groningen, Groningen, Netherlands; 7 Division of Biodeterioration and Reference Organisms, Federal Institute for Materials Research and Testing (BAM), Berlin, Germany; 8 Faculty of Medicine, University of Southampton, Southampton, SO17 1BJ UK; 9 Centre for Hybrid Biodevices, University of Southampton, Southampton, SO17 1BJ UK; 10 Centre for Proteomics Research, University of Southampton, Southampton, SO17 1BJ UK

**Keywords:** Pathogens, Biofilms, Antimicrobials

## Abstract

*Pseudomonas aeruginosa* MPAO1 is the parental strain of the widely utilized transposon mutant collection for this important clinical pathogen. Here, we validate a model system to identify genes involved in biofilm growth and biofilm-associated antibiotic resistance. Our model employs a genomics-driven workflow to assemble the complete MPAO1 genome, identify unique and conserved genes by comparative genomics with the PAO1 reference strain and genes missed within existing assemblies by proteogenomics. Among over 200 unique MPAO1 genes, we identified six general essential genes that were overlooked when mapping public Tn-seq data sets against PAO1, including an antitoxin. Genomic data were integrated with phenotypic data from an experimental workflow using a user-friendly, soft lithography-based microfluidic flow chamber for biofilm growth and a screen with the Tn-mutant library in microtiter plates. The screen identified hitherto unknown genes involved in biofilm growth and antibiotic resistance. Experiments conducted with the flow chamber across three laboratories delivered reproducible data on *P. aeruginosa* biofilms and validated the function of both known genes and genes identified in the Tn-mutant screens. Differential protein abundance data from planktonic cells versus biofilm confirmed the upregulation of candidates known to affect biofilm formation, of structural and secreted proteins of type VI secretion systems, and provided proteogenomic evidence for some missed MPAO1 genes. This integrated, broadly applicable model promises to improve the mechanistic understanding of biofilm formation, antimicrobial tolerance, and resistance evolution in biofilms.

## Introduction

*Pseudomonas aeruginosa* is a Gram-negative bacterium ubiquitously present in the soil, water, and different animal hosts^1^. As an opportunistic human pathogen^2^, it can cause sepsis, and chronic wound and lung infections, especially in immunocompromised and cystic fibrosis patients. Two mechanisms complicate the treatment of *P. aeruginosa* infections. It forms recalcitrant biofilms in which the bacterial cells have an increased tolerance against antimicrobial compounds^3,4^. In addition, worldwide, multiple genetic variants have acquired antimicrobial resistance (AMR) traits^5^, either through the acquisition of resistance genes on mobile genetic elements such as plasmids^6^ or through de novo mutations of chromosomal genes^7^. Furthermore, mutations affecting outer membrane porins and multidrug efflux pumps can mediate resistance to almost all major antibiotic classes and several important biocides^8,9^. *P. aeruginosa* thus also belongs to the notorious group of ESKAPE pathogens, which represent the leading causes of worldwide nosocomial infections (*Enterococcus faecium*, *Staphylococcus aureus*, *Klebsiella pneumoniae*, *Acinetobacter baumannii*, *P. aeruginosa*, and *Enterobacter* species)^10,11^. Clinically most relevant are the resistances of *P. aeruginosa* strains against fluoroquinolones, aminoglycosides, and beta-lactams, and against the last-resort antibiotic colistin (polymyxin). In 2017, the World Health Organization (WHO) classified carbapenem-resistant *P. aeruginosa* strains in the highest priority group of “critical pathogens”. New treatment options informed by a more detailed molecular understanding of how and why resistance emerges during treatment, and how resistance is transmitted, are urgently needed for such critical pathogens.

Increased antimicrobial tolerance, a fundamental property of biofilms^12^ is well-studied^13^ and four mechanisms have a major role: (i) under nutrient-limited conditions in biofilms, *P. aeruginosa* expresses phenotypic variants, i.e., dormant cells that are less susceptible to antibiotics which target actively dividing cells^14^; (ii) *P. aeruginosa* form a protective extracellular matrix composed of polysaccharides, proteins, and DNA that limits the diffusion of antimicrobial substances or sequesters them, such that biofilm cells experience a decreased antimicrobial dosage^15^; (iii) anoxic conditions exist within the biofilm limiting the efficacy of antibiotics that require aerobic metabolic activity and the generation of reactive oxygen species^16^; (iv) sub-inhibitory concentrations of antibiotics induce increased rates of mutation, recombination, and lateral gene transfer. The mutation rate in biofilms has been reported to be up to 100 times higher than in planktonic cells^17^, significantly accelerating the development of antibiotic-resistant mutants. Together, these mechanisms lead to hard-to-treat, chronic infections during which *P. aeruginosa* can persist and further evolve within the host in the presence of antimicrobial substances. Evolution within biofilms is highly parallel and differs significantly from the evolution of planktonic cells^18^. However, the evolutionary drivers of within-biofilm AMR evolution remain poorly understood. Their study requires well-defined model systems and tools, including model strains with complete genomic background information, genetic tools, and flow chambers allowing representative and reproducible growth of *P. aeruginosa* biofilms and deep sequencing data^18^.

The canonical reference model strain for *P. aeruginosa* is PAO1, also referred to as PAO1-UW. Its complete genome sequence was published in 2000^2^, which allowed many breakthrough discoveries. However, a number of closely related PAO1 strains exist that differ in their phenotypic appearances^19^. These include *P. aeruginosa* strain MPAO1^20^, the parental strain of the widely utilized transposon insertion mutant library from the University of Washington (UW)^21^. Such mutant collections represent highly valuable resources to uncover new functions and condition-specific essential genes in genome-wide screens^21^, for example, genes relevant for resistance against certain antibiotics^22,23^. They have also been used to define so-called general essential genes, i.e., genes that were identified as essential under more than one relevant growth condition^24,25^. As a subset of the conserved core genes *of P. aeruginosa* PAO1 and PA14 were shown to exhibit differential essentiality^25^, the approach to focus on those general essential genes that are furthermore conserved among key pathogen strains of a species is particularly promising^26^. However, the utility of such libraries to identify gain of function mutations is limited and polar effects need to be controlled for^27^. Notably, no complete MPAO1 genome sequence was available. Improvements in next-generation sequencing (NGS) technologies^28^ and assembly algorithms nowadays allow researchers to readily generate complete de novo genome assemblies for most prokaryotes except a few percent of strains with highly complex repeat regions^29^. Such fully resolved genomes are advantageous compared to fragmented short read-based genome assemblies that can sometimes even miss conserved core genes^30^; they are an ideal basis for subsequent functional genomics and systems biology studies and allow researchers to identify so far missed genes in genome annotations by proteogenomics^31^.

Here, we set out to develop, validate, and make available a model system to study the biofilm-associated adaptation to antimicrobials and AMR evolution in *P. aeruginosa* MPAO1. Conceptually, the model was designed to integrate genotype information with phenotypic data and to leverage the valuable genetic tools and wealth of functional genomics data sets that exist for important bacterial model organisms. Important elements include the complete MPAO1 genome sequence and the design for a standardized flow chamber based on accessible soft lithography replication in poly(dimethylsiloxane) (PDMS) that can deliver laminar flow conditions relevant to typical biofilm niches. Comparative genomics with the PAO1-UW reference strain uncovered numerous MPAO1-unique genes. Strikingly, these included 39 essential genes that had been missed so far by performing reference-based mapping of public Tn-seq data sets. Proof of principle experiments highlighted reproducible biofilm growth using the microfluidic flow chamber and identified hitherto unknown genes important for biofilm growth and biofilm-associated AMR through microtiter plate screening of the mutant library. A differential (planktonic vs. biofilm) proteomic data set uncovered gene products known to have a role in biofilm formation. Finally, a publicly available, integrated proteogenomics search database enables the identification of unannotated genes in MPAO1.

## Results

### De novo genome assembly of MPAO1

The availability of a complete genome sequence is an important pre-requisite to study the phenotypic adaptation and evolution of resistance to antimicrobials in biofilms. An analysis of over 9300 completely sequenced, publicly available bacterial genomes^29^ (see “Methods” section) listed 106 *P. aeruginosa* strains overall, two of which were *P. aeruginosa* PAO1 strains, including the PAO1 type strain (Genbank AE004091), also called PAO1-UW^2^. In contrast, the only strain annotated as MPAO1, i.e., the founder strain of the transposon mutant library available from the UW^21^, had been sequenced with Illumina short reads, assembled into 140 contigs^32^ and deposited (http://www.pseudomonas.com/strain/show?id=659; Genbank ASM24743v2) in the *Pseudomonas* genome DB^33^. To provide an optimal basis for subsequent functional genomics and evolution studies for *P. aeruginosa* strain MPAO1, we thus first sequenced and de novo assembled its complete genome. Due to the genomic differences reported for MPAO1 and PAO1^20^ and the fact that many of the 106 completely sequenced *P. aeruginosa* strains have difficult to assemble genomes with long repeat pairs in excess of 10 kilobases (kb) (38/106), so-called class III genomes^29^, we used third-generation long reads from Pacific Biosciences’ (PacBio) RSII platform. By relying on size-selected fragments (average length 9 kb; see “Methods” section), a single bacterial chromosome could be assembled. Additional genome polishing steps with Illumina MiSeq data (300 bp, PE reads) allowed the removal of remaining homopolymer errors in the PacBio assembly^34^. The final, high-quality MPAO1 genome consisted of one chromosome of 6,275,467 bp and coded for 5926 genes (Genbank CP027857; Table 1). An overview of selected predicted genome features (see “Methods” section) is shown in Supplementary Table 1. To facilitate data mining and comparison, we also provide an extensive annotation of all 5799 protein-coding genes. This includes information on conserved and MPAO1-unique genes compared to PAO1, the respective reciprocal best BLAST hits, protein domains, families, Gene Ontology (GO) classification, predictions of subcellular localization, lipoproteins, secreted and described membrane-localized proteins, as well as gene essentiality status and protein abundance data below (Supplementary Data 1).

**Table 1:** Summary over the core and strain-specific CDS of strains MPAO1 and PAO1-UW.

Category	*P. aeruginosa* MPAO1	*P. aeruginosa* PAO1-UW
Total no. of genes	5926	5697
Total no. of CDS	5799	5572
No. of core CDS (clusters^a^)	5548 (5534)	5545 (5534)
No. of unique (strain-specific) CDS (clusters)	234 (232)	19 (21)
Unique ncRNA	–	3
CDS ≤ 120 bp^b^	17	5

^a^All individual CDS are shown including those that are grouped in gene clusters (paralogs) in Fig. 1c.

^b^CDS of 120 bp or below are not considered (see “Methods” section).

### Comparative genomics of MPAO1 and PAO1 strains

**Fig. 1:**
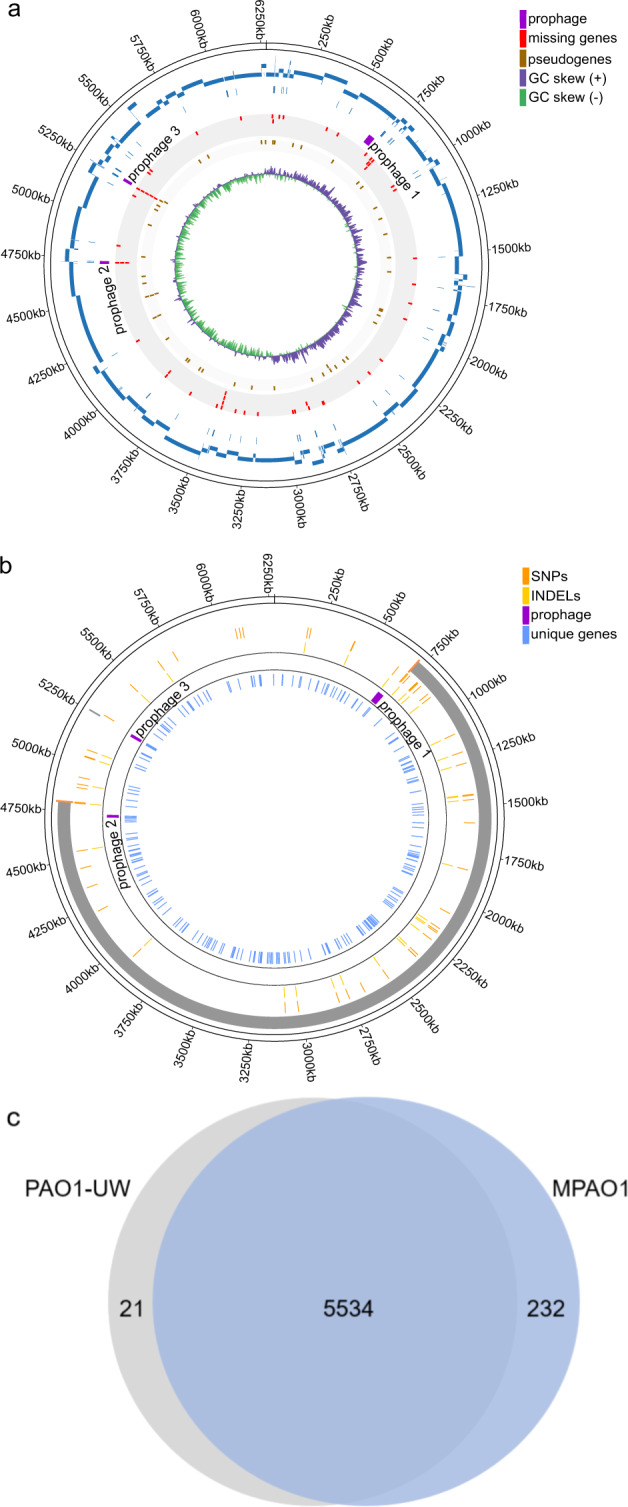
Genome map of *P. aeruginosa* MPAO1 and comparison to other strains. **a** The Circos plot visualizes the comparison of our complete MPAO1 genome (outer circle with genome coordinates) and that of strain MPAO1/P1 (second circle; blue), the respective gaps (third circle; blue) followed by annotated prophages (fourth circle; purple), missing genes (fifth circle, red), pseudogenes (sixth circle; brown), and GC skew (seventh circle; positive - purple; negative - green). **b** Differences of the MPAO1 genome compared to the PAO1 reference strain. Going from outer towards inner circles, the following genome features are shown: (1) a large inversion (gray) flanked by rRNAs (not shown), (2) SNPs (dark orange), (3) INDELs (light orange) (4) prophages (purple), (5) genes unique to MPAO1 (blue). **c** Comparative genomic analysis of *P. aeruginosa* strains MPAO1 and PAO1-UW. The Venn diagram shows the core gene clusters (paralogous genes are grouped into the same cluster provided they belong to a syntenic genomic region) and the respective number of strain-specific CDS clusters.

An alignment of our de novo assembled MPAO1 genome with that of the MPAO1/P1 strain^32^ revealed that overall, 42,813 bp of our complete genome sequence were missed by the 140 contigs of the available fragmented Illumina assembly (Fig. 1a). This comprised 66 genes (52 protein-coding genes, (CDS)) either missed completely or partially, including eight of 12 rRNA genes (75%) and six of 63 tRNA genes (11%). Among the CDS, the essential gene *ftsY* encoding the signal recognition particle-docking protein FtsY was missing, four of eight (50%) non-ribosomal peptide synthetase (NRPS) genes, three of six (50%) filamentous hemagglutinin N-terminal domain protein-coding genes, and three of 10 (30%) type VI secretion system (T6SS) VgrG effector proteins (Supplementary Data 1). The analysis of the number of interrupted genes or pseudogenes also confirmed the fragmented nature of the MPAO1/P1 genome compared to the complete genomes of both our assembly and the PAO1-UW type strain (Supplementary Fig. 1). Importantly, a key study of the genotypic and phenotypic diversity of *P. aeruginosa* PAO1 strains recently reported 10 PAO1/MPAO1 laboratory isolates as complete genomes^19^. As all 10 genomes have been assembled using Illumina data into sets of contigs, strictly speaking, they are not fully assembled, closed genome sequences. Indeed, the genomes of the two MPAO1 strains in that list (PAO1-2017-E, 71 contigs, whole genome shotgun (WGS) QZGA00000000 and PAO1-2017-I, 70 contigs, WGS QZGE00000000) also lacked a similar amount of genomic sequence (56.5 and 59.4 kb) and number of genes (55, 62) or CDS (40, 47) respectively, compared to our complete genome (Supplementary Data 1).

Next, to explore the extent of strain-specific genomic differences, we created an alignment of our de novo assembled MPAO1 genome with that of *P. aeruginosa* strain PAO1-UW. This analysis confirmed the major differences reported previously^20^, i.e., the presence of a third prophage region (12.8 kb, 20 genes; genome coordinates 5,241,813–5,254,613) in strain MPAO1 (Fig. 1b) and the absence of a ~1 kb genome fragment (leading to a pseudogene annotation for MPAO1_24940 in MPAO1). An analysis of smaller differences between the genomes confirmed the 16 SNPs reported previously^20^ and identified 176 additional SNPs and INDELs between MPAO1 and PAO1 that had not been reported by Klockgether and colleagues^20^ (Supplementary Data 2).

Notably, while the overall number of predicted genes was close for both strains (Table 1), we observed 232 gene clusters specific to strain MPAO1 and 21 clusters specific to strain PAO1-UW (Fig. 1c), suggestive of potentially relevant differences between the strains. The annotation of the shared (core) and strain-specific (unique) gene clusters is provided in Supplementary Data 3. This analysis indicated that a sizeable set of genes were specific to the MPAO1 genome and that mapping data sets obtained from this strain back to the PAO1-UW genome could overlook important genes (see below). A gene ontology (GO) enrichment analysis of the MPAO1-unique proteins against all CDS in its genome revealed that the biological process “protein phosphorylation” was significantly enriched (*p* value < 0.01) with 10 hits among all genes including three among the unique genes (including a DNA helicase and 2 serine/threonine protein kinases; Supplementary Table 2). Furthermore, for the biological process “Bacteriocin immunity” five hits were found among all genes, two of which were among the unique MPAO1 genes (Supplementary Table 2).

### Tn-seq data mapping

The complete MPAO1 genome sequence allowed us to re-analyze public Tn-seq data sets without the limitation of any remaining “genomic blind spots” that otherwise might preclude identification of all essential genes^26^, and the drawbacks of mapping Tn-seq data to a closely related reference genome. A re-mapping of MPAO1 Tn-seq data sets obtained from several conditions (LB medium, minimal medium, sputum, and brain-heart infusion BHI medium)^24^ against both the PAO1-UW genome and our MPAO1 genome (see “Methods” section), confirmed our expectation. We indeed observed a higher percentage of mapped reads for MPAO1 (roughly 0.1–0.35% of all mapped reads per sample; Supplementary Table 3) and unique insertion sites (roughly 0.2% more in MPAO1, Supplementary Table 3). Genes with no insertion or genes whose *p*-value was less than 0.001 were considered essential (see “Methods” section). Overall, 577 genes were classified as condition-specific essential in one of the three primary growth conditions LB medium, minimal medium, sputum (Supplementary Data 4), and 312 genes represented general essential genes, i.e., were essential in all three growth conditions, respectively (Supplementary Fig. 2). Importantly, close to 40 MPAO1 unique genes were linked here with an essentiality status, as they were essential in one or more of the 16 Tn-seq libraries (Supplementary Data 4). By mapping data against the PAO1-UW genome, these genes had been previously overlooked in the analysis of essential *P. aeruginosa* genes.

**Fig. 2:**
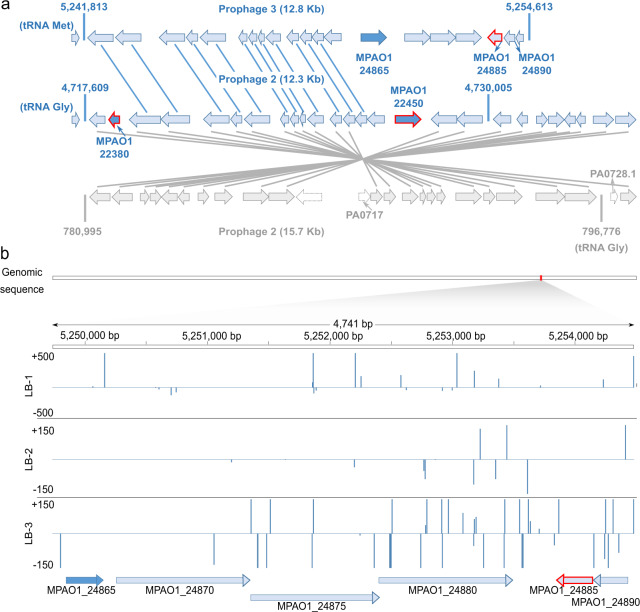
An overview of annotated genes in selected prophage regions and their essentiality classification. MPAO1-unique essential genes are shown in dark blue, general essential MPAO1 genes with a red arrow outline. **a** Genes located in prophage region 2 of PAO1-UW (gray), the corresponding inverted region in strain MPAO1 (light blue arrows in middle), and the prophage region 3 (light blue arrows on top) unique to MPAO1 are shown (not drawn to scale), the genomic positions of their boundaries (5′–3′) and flanking tRNAs. Genes connected by lines are orthologous to each other based on comparative genomics combined with a Blast analysis. **b** Transposon insertions in selected genes of prophage region 3 of MPAO1. Insertion frequencies in six genes are shown using data mapped from the LB-1 (3 replicates), LB-2 (2 replicates), and LB-3 (1 sample) Tn-seq libraries. Non-essential genes (based on a data set of 577 genes essential in one of three primary growth conditions) are shown in light blue.

Among these MPAO1-unique genes, we identified 18 genes that were essential in 50% or more of the Tn-seq runs, six of which represented general essential genes (Table 2). The general essential genes included two genes located in the prophage 2 region, i.e., MPAO1_22380, a type II Phd/YefM family antitoxin gene located next to MPAO1_22375, coding for a RelE/ParE type toxin, and MPAO1_22450, a DNA-binding protein (Fig. 2a; arrows framed in red). A further general essential gene was MPAO1_00215 encoding for a hypothetical protein. MPAO1_00215 is located in a genomic region that harbors another essential gene (MPAO1_00230, Supplementary Data 1), that may represent an operon.

**Table 2:** List of 18 selected MPAO1-unique genes along with their essentiality classification in all 16 Tn-seq samples^24^ and comments about their genomic location.

Locus	Gene annotation	General essential	Essential in x/16 samples	Comment
MPAO1_22380	Type II toxin-antitoxin system Phd/YefM family antitoxin	Yes	16	Prophage 2
MPAO1_00215	Hypothetical protein	Yes	15	*Operon?
MPAO1_10410	Hypothetical protein	Yes	14	
MPAO1_22450	DNA-binding protein	Yes	14	Prophage 2
MPAO1_25260	Cytidine deaminase		12	
MPAO1_12950	Hypothetical protein	Yes	11	
MPAO1_00230	Hypothetical protein		10	*Operon?
MPAO1_20095	Hypothetical protein		10	
MPAO1_02335	Dihydropyrimidinase		9	
MPAO1_15010	6-*O*-methylguanine DNA methyltransferase		9	
MPAO1_15215	Amino acid permease		9	
MPAO1_18025	Ferredoxin		9	
MPAO1_02315	Oxidoreductase		8	
MPAO1_05695	Hypothetical protein	Yes	8	Bacteriocin (GO)
MPAO1_08710	DUF3304 domain-containing protein		8	
MPAO1_10195	Universal stress protein		8	
MPAO1_14380	Glycosyltransferase		8	
MPAO1_24865	Hypothetical protein		8	Prophage 3

Information about all MPAO1-unique essential genes is available in Supplementary Data 4.

Furthermore, the prophage 3 region unique to strain MPAO1, harbored a gene encoding a hypothetical protein (MPAO1_24865; Fig. 2b) that was essential in eight of 16 samples (Table 2). Conversely, MPAO1_24885 (addiction module antidote protein from the HigA family toxin-antitoxin (TA) system) from this region was classified as general essential (Table 2; 14 of 16 samples). Due to its homology to PA4674 in PAO1-UW, which is listed among the 352 general essential genes reported by Lee and colleagues and encodes the HigA antitoxin^24^, it is not unique to MPAO1 (Fig. 2b). Together with the non-essential MPAO1_24890 (plasmid maintenance system killer protein; most similar to RelE-like toxins of the type II TA system HigB), MPAO1_24885 encodes for a TA system. However, there is no homolog annotated for MPAO1_24890 in PAO1-UW. Therefore, due to this missing gene, the TA system was not identified in PAO1-UW. This finding again underlines the importance of having the actual and complete genome sequence to map functional data.

### Reproducible formation of MPAO1 biofilms

**Fig. 3:**
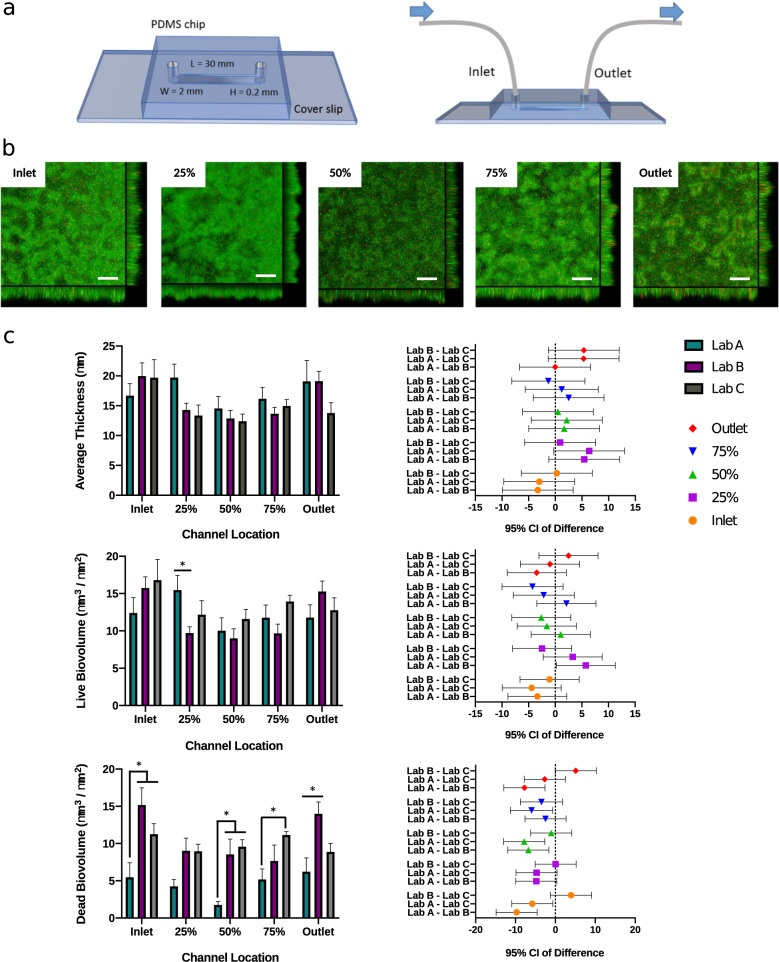
The publicly available mold design for the microfluidic flow chamber allows reproducible biofilm formation as confirmed by an inter-laboratory comparison. **a** Schematic and dimensions of the flow chamber. **b** Representative images of 72 h MPAO1 WT biofilms grown on the PDMS surface of the device under laminar flow conditions at five different locations along the channel. Biofilms were treated with live/dead staining (green – live cells stained with Syto9; red – dead cells stained with propidium iodide). Scale bar in confocal *XY* plane: 40 µm. Sagittal *XZ* section represents biofilm thickness. **c** COMSTAT data for average thickness, and live/dead biovolume of 72 h MPAO1 WT biofilms generated by three different laboratories, with 95% confidence interval comparisons (3 biological repeats comprising 3 technical repeats per site, i.e., *n* = 9 biological/*n* = 27 technical repeats overall; error bars - standard error of mean; 2-way ANOVA with lab and channel location as variables followed by multiple comparisons Tukey test). *p value < 0.05.

The second important objective of our integrated model system was to enable the reliable generation of phenotypic data under conditions relevant for biofilm growth. For this purpose, we focused on the development of a microfluidic flow chamber for reproducible biofilm formation that would allow us to subsequently identify genes relevant for biofilm growth and biofilm-associated AMR. The flow chamber was designed in such a way that we could assess the effects of hydrodynamic conditions^35^, such as shear stress and controlled flow conditions. Our flow chamber was replicated in PDMS, a simple to use, transparent, and breathable elastomer material that naturally adheres to the glass. A straight microfluidic channel design was used (30 mm length × 2 mm width × 0.200 mm depth) (Fig. 3a, see “Methods” section for further details). PDMS was selected due to its broad application in indwelling devices and implant materials^36^. The inlet and outlet of the microfluidic flow chamber comprised of sterile polytetrafluoroethylene (PTFE) tubing, a material that was chosen because it generally exhibits low bacterial adhesion. A syringe pump was used to deliver 5 μL/min (*ū* ≈ 208 µm/s) flow inside the chamber to provide laminar flow conditions for bacterial adhesion and biofilm growth (the calculated Reynolds Number corrected for the transport of water at 37 °C was 0.103; for details see Supplementary Table 4).

The reproducibility of a 72 h mature MPAO1 biofilm on the PDMS surface of the device was investigated by confocal laser scanning microscopy (CLSM) combined with live/dead staining using the dyes Syto9 and propidium iodide in three separate consortium laboratories all using the same microfluidic chamber mold (design publicly available; see Data availability) (Fig. 3b, c). The biofilms formed in the three laboratories were consistent with data falling within 95% confidence intervals, the only difference being the observation of a reduced dead biovolume in one laboratory’s model (Lab A; *p* value < 0.05). Biofilm formation was relatively uniform throughout the flow channel with an average thickness of 16 µm and a small reduction observed towards the center of the channel (Inlet—18.8 µm, 25%—15.8 µm, 50%—13.3 µm, 75%—14.9 µm, Outlet—17.3 µm). An average biovolume of 12.5 µm^3^/µm^2^ and dead biovolume of 8.4 µm^3^/μm^2^ was observed, again reducing towards the center of the device commensurate with the average biomass.

### Screening experiments identify known and new genes relevant for biofilm formation and antibiotic resistance

**Fig. 4:**
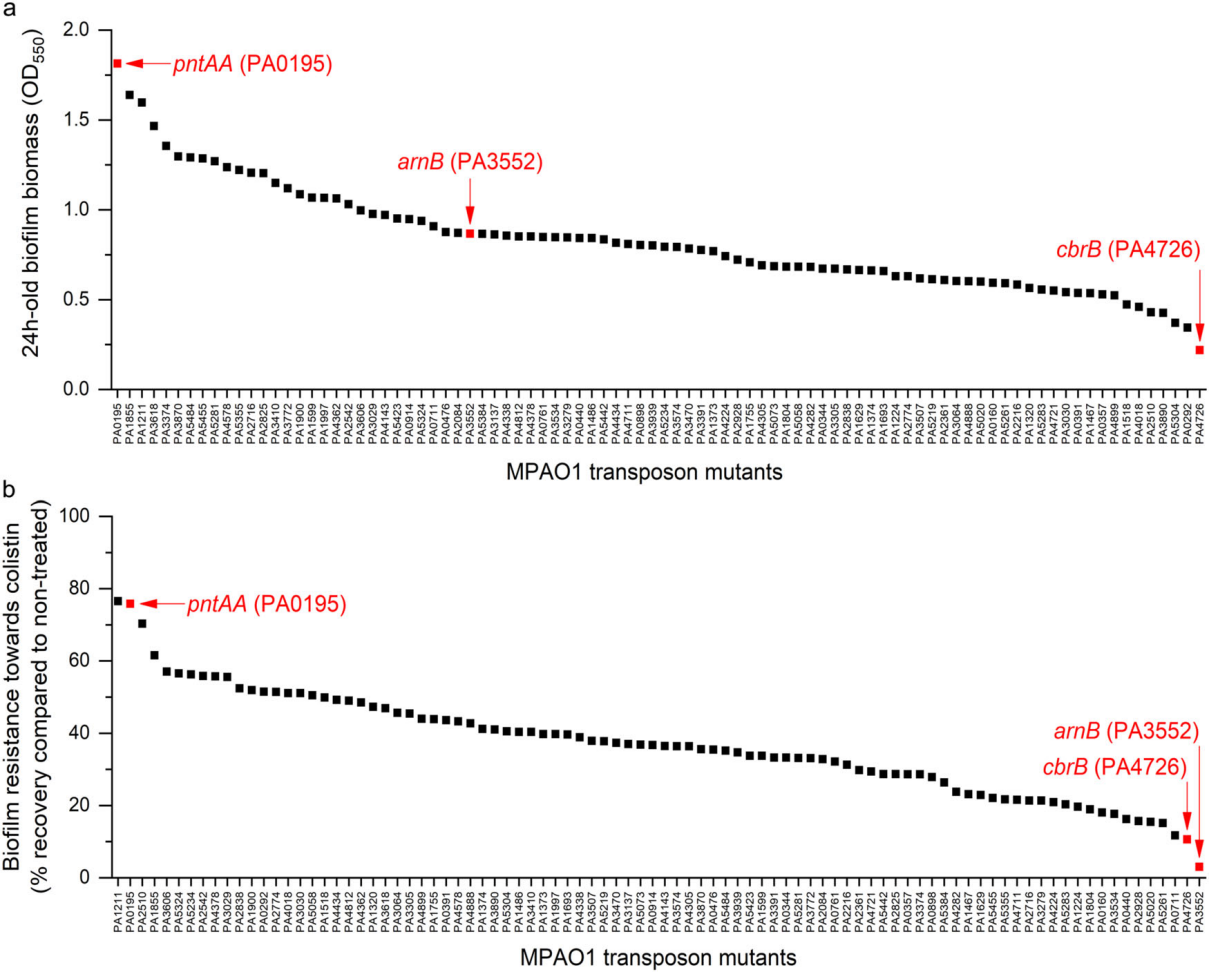
Proof of principle that biofilm growth-relevant and AMR-related genes can be identified in adequate screens using the MPAO1 transposon mutant library. A diagram of the protocol is shown in Supplementary Fig. 3. **a** Biofilm formation of 90 MPAO1 mutant strains (*X* axis) after 24 h incubation in the M9 medium (average of two independent wells). Biofilm biomass was quantified by crystal violet. **b** Ability of biofilms formed by 90 MPAO1 mutant strains to recover after colistin treatment (see “Methods” section). The recovery of treated biofilm cells was normalized to the recovery of non-treated biofilm cells (defined as 100%). The *arnB* mutant (PA3552) is highlighted in red, as well as the highest biofilm former missing *pntAA* (PA0195) and the lowest biofilm former missing *cbrB* (PA4726).

The MPAO1 transposon mutant library was tested with a 96-well plate screening system that was devised to enable the identification of genes that affect biofilm formation and/or have a role in the development of biofilm-associated AMR (see Supplementary Fig. 3). A batch of 95 selected mutants (Supplementary Table 5) was taken from the library to test the reliability of our protocol and to identify genes related to biofilm formation (in duplicate). Strain PW8965 harboring an insertion in *cbrB* (PAO1 identifier PA4726, MPAO1_25185), a transcriptional activator that forms part of the CbrA/CbrB two-component system important in catabolite repression^37^, was found to produce the least amount of biofilm (Fig. 4a). In contrast, strain PW9283 mutated in *pntAA* (PA0195; MPAO1_01040), an NADPH/NAD^+^ redox balance transhydrogenase^38^, exhibited the highest biofilm biomass.

**Fig. 5:**
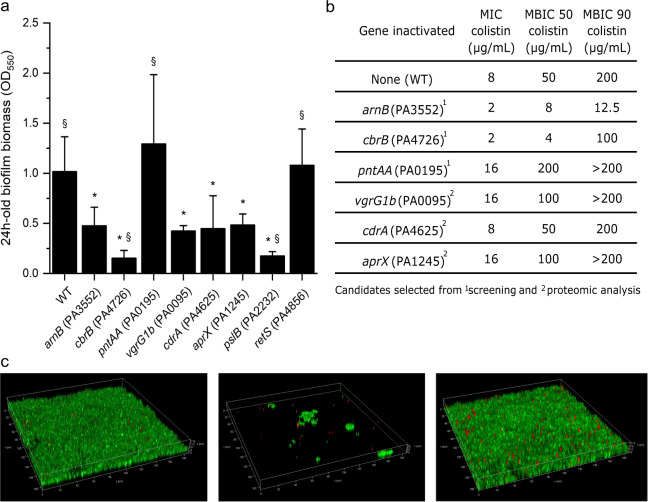
Confirmation of the phenotypes identified in our screening. **a** Biofilm formation was quantified after 24 h incubation in M9 medium by crystal violet staining (average of at least 18 wells from two independent cultures). The *pslB* and *retS* mutants were used as a reference for low and high biofilm formation, respectively. The *cbrB* and *pntAA* mutants demonstrated substantially reduced and increased biofilm formation, respectively. Symbols (* and ^§^) indicate significant differences (Student’s tests with *p* value < 0.001) in comparison to MPAO1 WT and the *arnB* mutant, respectively. PAO1 genes are shown in brackets, the respective MPAO1 genes are mentioned in the text. **b** Resistance of planktonic and biofilm cells towards colistin was evaluated for a subset of mutant strains identified in the screening (^1^) or based on differential proteomics abundance (^2^). The MIC was determined as the lowest concentration resulting in a 90% reduction of bacterial growth after 24 h in M9 medium compared to the non-treated condition (average of four replicates from two independent cultures). The MBIC was determined as the lowest concentration resulting in a 50% or 90% reduction of the biofilm cell recovery after 24 h treatment compared to the non-treated condition (average of four replicates from two independent cultures). **c** Comparative confocal micrographs after live/dead staining (green – live cells stained with Syto9; red – dead cells stained with propidium iodide) of 18 h MPAO1 WT, *cbrB* and *arnB* biofilms grown under microfluidic conditions using the publicly available mold confirm reduced biofilm formation for the *cbrB mutant* and robust biofilm formation of the *arnB mutant* in the absence of treatment.

In a second step, selected mutants identified by the screening and the proteomic analysis (see below) were compared to positive and negative controls for biofilm formation (Fig. 5a). The *pslB mutant* (PA2232; MPAO1_14370), a gene whose product is involved in the synthesis and export of polysaccharides, was used as a reference point for low biofilm formation^39^, while a *retS* mutant (PA4856; MPAO1_25880), encoding a pleiotropic regulator of multiple virulence factors, was used as a reference point for high biofilm formation^40^. Overall, MPAO1 WT produced roughly twice the biofilm biomass of transposon mutants, suggesting that the transposon has an influence on biofilm formation and that it is more reliable to compare transposon mutants amongst each other. The transposon mutant PW7021 (an *arnB* mutant; PA3552; MPAO1_07345, see below) was chosen as an internal reference for biofilm formation as its biomass was found approximately midway through the 24 h biofilm readings in Fig. 4a. We confirmed that the *cbrB* mutant produced significantly less biofilm biomass (*p* value < 0.001) than the *arnB* mutant, similar to the low biofilm-forming *pslB* mutant. Biofilm growth of the *cbrB* mutant was also performed within the flow chamber to confirm the capacity of the device to assess differential biofilm formation. Similar to the 96-well plate screening assay, the *cbrB* mutant produced substantially less biofilm compared to the MPAO1 WT over 18 h in the flow chamber **(**Fig. 5c) and displayed a delayed exponential growth compared to WT and the other mutants tested (Supplementary Fig. 4). We also confirmed that the *pntAA* mutant produced higher biofilm biomass than other transposon mutants, similar to the high biofilm former *retS* mutant (Fig. 5a). However, compared to the WT, the *retS* mutant produced comparable biofilm biomass, which is likely caused by a decrease of strain fitness due to the transposon insertion. An alternative explanation is that the effect of RetS cannot be measured after 24 h because it has been shown previously that RetS turns non-functional in *P. aeruginosa* WT after 8 h following initial attachment^41^. Genes identified by the proteomic analysis (*vgrG1b*, *cdrA*, *aprX*; see “Result” section below) did not seem to affect the biofilm formation of MPAO1 in the conditions tested.

Next, we tested the strains for their biofilm resistance to colistin and included the *arnB* mutant strain PW7021 as a positive control (Supplementary Fig. 3). ArnB is a well-studied protein known to modify lipopolysaccharide (LPS) and have a key role in the resistance to colistin^42,43^. The recovery of biofilm cells after treatment with 25 µg/mL colistin was compared to the recovery of non-treated biofilm cells (Fig. 4b) (see “Methods” section), as described previously^13^. This concentration of colistin was much higher than the minimal inhibitory concentration (MIC) used for the planktonic *P. aeruginosa* MPAO1 (8 µg/mL) allowing us to focus specifically on biofilm cells. As expected, the *arnB* mutant exhibited a very low recovery after colistin treatment (97% less than the control without colistin) (Fig. 4b). In contrast, the *arnB* mutant produced robust biofilms in the biofilm screening assay (Fig. 4a), a phenotype that was confirmed using the microfluidic chamber (Fig. 4c). Notably, the *cbrB* mutant strain grown as a biofilm was also found to be sensitive to colistin (90% less recovery than the control without colistin; Fig. 4a), which might be related to the low amount of biofilm produced by this mutant. In contrast, the high biofilm former *pntAA* mutant exhibited high resistance towards colistin with recovery close to the non-treated biofilm.

In a second step, the resistance profile of the identified mutants was characterized in more detail by measuring the MIC of planktonic cells and the minimal biofilm inhibitory concentration (MBIC) towards colistin (Fig. 5b). Two independent experiments (with four replicates in total) confirmed the significantly higher sensitivity of planktonic cells of *arnB* and *cbrB* mutants compared to the WT (Fig. 5b). Additionally, inactivation of the genes *arnB* and *cbrB* reduced the biofilm recovery by 50% when 6 and 12 times less colistin was used, respectively, compared to the WT. Inactivating *cbrB* made MPAO1 biofilms more sensitive to low concentrations of colistin, but high concentrations seemed necessary to reach complete eradication (Fig. 5b). In contrast, inactivating *pntAA* increased *P. aeruginosa* resistance towards colistin both as planktonic and biofilm cells. Characterization of the genes identified in the proteomic study (see below) revealed that inactivating *cdrA* had no impact on MPAO1 resistance, but inactivation of *vgrG1b*^44^ and *aprX*^45^ increased MPAO1 resistance towards colistin both for planktonic and biofilm cells (Fig. 5b).

### Protein abundance profiling of MPAO1 grown planktonically and in biofilms

**Fig. 6:**
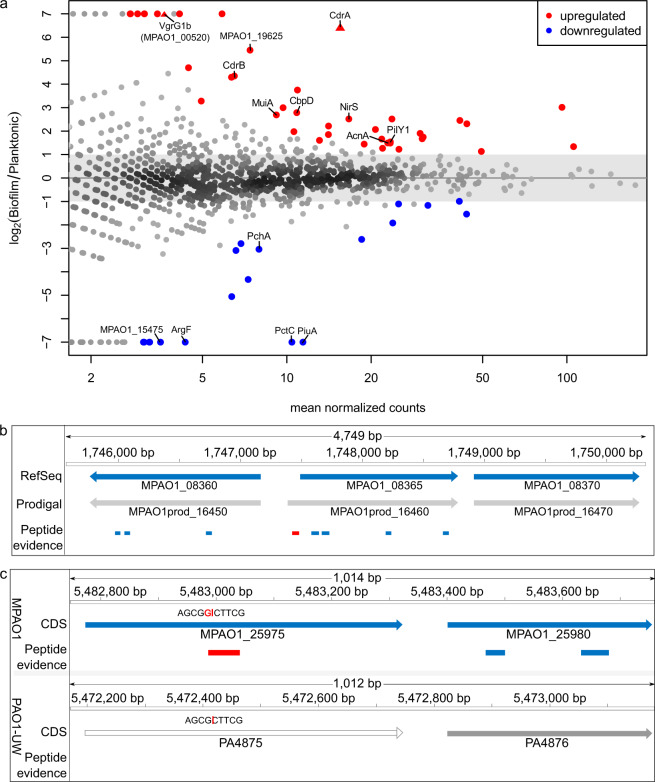
Proteomic experiments identify known biofilm-related proteins and new information. **a** Differential protein abundance between MPAO1 mid-exponential planktonic cells and 72 h biofilms. Selected significantly upregulated proteins (red dots) known to have a role in biofilm formation/growth are labeled, proteins downregulated in planktonic growth are shown in blue. Red triangles denote proteins encoded by genes missed in the MPAO1/P1 genome. **b** Proteogenomic expression evidence for a longer protein than annotated by RefSeq: the Prodigal predicted protein MPAO1prod_16460 (gray arrow; 447 aa; amino acid) is 44 aa longer than the RefSeq annotated MPAO1_08365 and encodes a glutamine synthetase (blue arrow; 413 aa). The NH-terminal extension is supported by 1 peptide (red) with seven PSMs and harbors a 40 aa longer glutamine synthetase N-terminal domain compared to the RefSeq protein. **c** Proteogenomic expression evidence for a single nucleotide insertion (red) in the MPAO1_25975 gene (blue arrow) compared to its PAO1 homolog PA4875 (annotated as pseudogene; gray open arrow). The change is supported by peptide evidence (1 red bar).

To assess if we could identify proteins known to have a role in biofilm formation with the microfluidic chamber, we next generated shotgun proteomics data for MPAO1 cells grown to mid-exponential planktonic phase or as 72 h biofilms (3 replicates each). 1530 and 1728 proteins were identified in planktonic cells and biofilm, respectively, resulting in a combined 1922 of the 5799 annotated proteins (33.1%). Among the most significantly differentially abundant proteins (log_2_ fold change (FC) of ≥ 1 or ≤−1 and adjusted *p* value ≤ 0.05; see “Methods” section and Supplementary Fig. 5) several candidates were identified that have previously been linked with a role in biofilm formation. These included MuiA (MPAO1_18330)^46^, CbpD (MPAO1_21730)^47^, AcnA (MPAO1_17965)^48^ and PilY1 (MPAO1_24155)^49^ (Fig. 6a, Table 3; see “Discussion” section). In addition, MPAO1_19625 was highly upregulated in biofilms (Fig. 6a). Notably, its PAO1 homolog AprX was reported to be secreted by a type I secretion system^45^, indicating that hypothetical proteins or proteins of unknown function can be linked to roles in biofilm formation and growth. We next looked for protein expression evidence for CDS missed in the fragmented short-read genome assemblies. We found that 21 of the 52 CDS missed in the MPAO1/P1 assembly were detected at the protein level (Supplementary Data 1). Notably, this included two proteins significantly upregulated in the biofilm, namely MPAO1_00520 (T6SS tip protein VgrG1b) located close to the H1-T6SS cluster^44^ and MPAO1_24535 (Fig. 6a), the homolog of PAO1 CdrA, a cyclic-di-GMP-regulated adhesin known to reinforce the biofilm matrix^50^, again underlining the importance of a complete genome sequence for downstream functional genomics analyses. Notably, nine of 14 structural genes of H1-T6SS, one of overall three T6SSs in *P. aeruginosa* that helps it to prevail under stressful conditions^51^, were upregulated around two-fold or more in biofilm (Supplementary Fig. 6). Similarly, all three VgrG1 proteins (1a-1c) that are co-regulated with the H1-TS66^52^ were upregulated in biofilm, while none of the other seven VgrG family members were expressed. Among the proteins downregulated in 72 h biofilms, three are associated with the iron acquisition; isochorismate synthase (MPAO1_03800), a rate-limiting enzyme involved in the production of salicylate (precursor of the siderophore pyochelin)^53^, the siderophore receptor MPAO1_23930 (PuiA), and the siderophore-interacting protein MPAO1_15475. Iron acquisition is deemed necessary for *P. aeruginosa* biofilm formation^54^ so their down-regulation was unexpected, however, this response is likely circumvented by the utilization of alternative iron acquisition strategies including the high-affinity siderophore pyoverdine.

**Table 3:** List of 61 proteins with significant differential abundance (see text) or unique expression when comparing biofilm grown and planktonic cells.

Locus tag	Gene	Product	log2 FC	*p*_adj_	Comment, reference
Biofilm only					
MPAO1_19985	*napA*	Nitrate reductase catalytic subunit NapA	5.02	0.05	
MPAO1_04195		SH3 domain-containing protein	5.02	0.05	
MPAO1_10705		Methyl-accepting chemotaxis protein	5.11	0.03	
MPAO1_17160		EscC/YscC/HrcC family-type III secretion system outer membrane ring protein	5.11	0.03	
MPAO1_21585		Itaconyl-CoA hydratase	5.19	0.04	
MPAO1_17195		Translocator outer membrane protein PopD	5.19	0.02	
MPAO1_17200		Hypothetical protein	5.34	0.01	
MPAO1_00520	vgrG1b*	**Type VI secretion system tip protein VgrG1b**^52^	**5.41**	**0.01**	H1-T6SS^44^
MPAO1_20935		Beta-keto-ACP synthase	5.61	0.04	
MPAO1_24325		Cytochrome c551 peroxidase	6.11	0.00	
Diff. Abundant					
MPAO1_07815		Osmoprotectant NAGGN system M42 family peptidase	4.70	0.02	
MPAO1_19625	*aprX*	Hypothetical protein	5.45	0.00	^45^
MPAO1_24535	cdrA*	**Filamentous hemagglutinin N-terminal domain-containing protein**	**6.54**	**0.00**	^50^
MPAO1_02725	*nirF*	Protein NirF	4.30	0.01	
MPAO1_24530	cdrB*	ShlB/FhaC/HecB family hemolysin secretion/activation protein	4.35	0.01	^50^
MPAO1_25250		BON domain-containing protein	3.28	0.05	
MPAO1_19595		Serralysin	3.75	0.01	
MPAO1_22090	*putA*	Bifunctional proline dehydrogenase/L-glutamate gamma-semialdehyde dehydrogenase PutA	3.00	0.01	
MPAO1_18330	muiA*	Mucoidy inhibitor MuiA	2.69	0.01	^46^
MPAO1_21730	cbpD*	Chitin-binding protein CbpD	2.79	0.00	^47^
MPAO1_06120		Copper chaperone PCu(A)C	1.98	0.03	
MPAO1_14990		NAD(P)-dependent alcohol dehydrogenase	2.22	0.01	
MPAO1_02740	*nirS*	Nitrite reductase	2.52	0.00	
MPAO1_25230		DUF748 domain-containing protein	1.85	0.02	
MPAO1_18000	*ccoP*	Cytochrome-c oxidase, cbb3-type subunit III	1.60	0.05	
MPAO1_28880	*adhP*	Alcohol dehydrogenase AdhP	2.52	0.00	
MPAO1_07010		Phosphoketolase	2.07	0.00	
MPAO1_00100		LysM peptidoglycan-binding domain-containing protein	1.44	0.03	
MPAO1_02290		TonB-dependent receptor	1.66	0.01	
MPAO1_27435		Amino acid ABC transporter substrate-binding protein	−3.09	0.03	
MPAO1_05385		DUF1302 domain-containing protein	−2.80	0.03	
MPAO1_17965	acnA*	Aconitate hydratase	1.49	0.01	^48^
MPAO1_24155	pilY1*	Type 4a pilus biogenesis protein PilY1	1.54	0.01	^49^
MPAO1_05375		Fatty acid--CoA ligase	−5.06	0.01	
MPAO1_04650		OmpW family protein	1.49	0.01	
MPAO1_00495	*tssH*	Type VI secretion system ATPase TssH	1.27	0.03	H1-T6SS^44^
MPAO1_14010		Non-ribosomal peptide synthetase (NRPS)	1.90	0.02	
MPAO1_26210	*azu*	Azurin	2.45	0	
MPAO1_13620		Xanthine dehydrogenase family protein molybdopterin-binding subunit	−4.33	0.01	
MPAO1_03800	*pchA*	Salicylate biosynthesis isochorismate synthase	−3.04	0.01	
MPAO1_06095		TonB-dependent copper receptor	1.74	0.00	
MPAO1_03775		Catalase	1.67	0.00	
MPAO1_02430	*clpG*	AAA family protein disaggregase ClpG	2.31	0.00	
MPAO1_26945		Poly(3-hydroxyalkanoate) granule-associated protein PhaF	1.22	0.03	
MPAO1_23990		Prepilin-type cleavage/methylation domain-containing protein	3.01	0.00	
MPAO1_02180		Response regulator	1.13	0.00	
MPAO1_05390		DUF1329 domain-containing protein	−2.62	0.00	
MPAO1_13900		NADP-dependent glyceraldehyde-3-phosphate dehydrogenase	−1.11	0.05	
MPAO1_13035		Multidrug efflux RND transporter periplasmic adaptor subunit MexE	−1.92	0.00	
MPAO1_25100		TonB-dependent hemoglobin/transferrin/lactoferrin family receptor	−1.17	0.02	
MPAO1_09260		Carbohydrate ABC transporter substrate-binding protein	−0.99	0.02	
MPAO1_16835		Porin	1.34	0.00	
MPAO1_09280		Porin	−1.54	0.00	
Planktonic only					
MPAO1_23930	puiA**	TonB-dependent siderophore receptor	−6.91	0.00	
MPAO1_22860	*pctC*	Methyl-accepting chemotaxis protein PctC	−6.78	0.00	
MPAO1_07425	*argF*	Ornithine carbamoyltransferase	−5.51	0.01	
MPAO1_21260		Chain-length determining protein	−5.22	0.02	
MPAO1_15475		Siderophore-interacting protein	−5.09	0.02	
MPAO1_29055		Class I SAM-dependent methyltransferase	−5.08	0.03	
MPAO1_22680		Biliverdin-producing heme oxygenase	−5.02	0.03	
MPAO1_09305	*pgl*	6-phosphogluconolactonase	−5.01	0.03	

Publications linking the genes/proteins with various roles in biofilms are listed for proteins highlighted in Fig. 6. Two genes missed in MPAO1/P1 are shown in bold. Gene names stem from the National Center for Biotechnology Information (NCBI) annotation or were deduced from the eggNOG annotation or the respective PAO1 homolog (*) or the *Pseudomonas* genome database (**); see also Supplementary Data 1.

Finally, to identify unannotated short ORFs that may carry out important functions or new start sites, we created an integrated proteogenomics search database (iPtgxDB) for strain MPAO1 and PAO1 (Supplementary Table 6), which cover their entire coding potential^31^. A search combined with stringent result filtering (see “Methods” section) allowed us to identify unambiguous peptide evidence^55^ for a 44 aa longer proteoform of MPAO1_08365 (predicted by Prodigal, an ab initio gene prediction algorithm; Fig. 6b). In addition, we obtained proteogenomic evidence supporting a single nucleotide insertion in MPAO1_25975 in strain MPAO1 as compared to PA4875 (annotated as pseudogene) in strain PAO1 (Fig. 6c). The peptide that supported this single nucleotide change at the amino acid level was identified with seven peptide spectrum matches (PSMs), illustrating the ability to identify SNP changes at the protein level, with implications for clinical proteomics.

## Discussion

*P. aeruginosa* is a member of the ESKAPE pathogens, the lead cause of worldwide nosocomial infections^10^. Along with many other clinically relevant bacteria, it can form biofilms whose emergent properties^56^ include a much higher tolerance to antimicrobials. Together with the increased mutation rates in biofilm compared to planktonic cells^17^, this further complicates treatment and cure of biofilm-based infections^12,13^. The development of model systems allowing the study of antimicrobial tolerance mechanisms and the evolutionary dynamics that lead to AMR development in biofilms is thus of utmost priority.

**Fig. 7:**
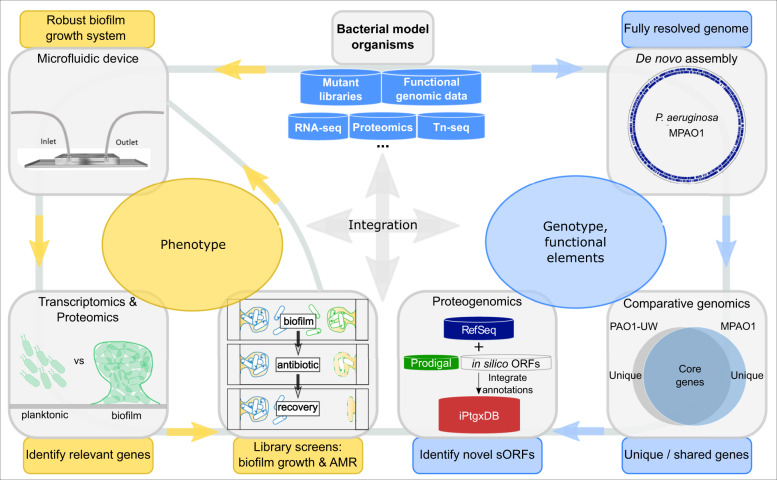
Integrated model system to identify and validate genes relevant for biofilm growth and AMR. A sequential genomics-driven workflow (blue arrows) to de novo assemble the complete genome, identify unique and conserved genes among key reference strains by comparative genomics and missed genes by proteogenomics is integrated with an experimental workflow in the form of an iterative cycle that can be entered at various points (yellow arrows). This workflow allows the study of biofilm grown cells, to explore differentially abundant genes or proteins compared to planktonic cells, and to screen mutant libraries to identify functionally relevant genes. The model leverages the enormous value of genetic resources like gene knockout or transposon insertion mutant libraries and functional genomics data sets (RNA-seq, Tn-seq, etc.; blue containers). Additionally, it allows for phenotypic characterization of biofilms formed by mutant strains, thereby allowing us to determine the impact of specific genes on biofilm formation and assess their role in AMR (yellow arrows).

We here develop and validate such a model system for *P. aeruginosa* MPAO1 (Fig. 7). Conceptually, the model was designed to integrate genotype data with phenotypic information and to leverage the wealth of existing public genetic resources and functional genomics data sets. A complete, fully resolved genome sequence is one critical element^31,57^, which recently allowed linking of genotypic differences of nine Pseudomonas plant microbiome isolates with their varying biocontrol potential^58^. While a complete genome existed for *P. aeruginosa* PAO1^2^, only three fragmented Illumina-based genome assemblies were available for MPAO1, the parental strain of the popular UW transposon mutant library^21^. These included strains MPAO1/P1^32^ and the recently sequenced PAO1-2017-E and PAO1-2017-I^19^. On average, they lacked between 55 to 66 genes (40–52 CDS) compared to our complete MPAO1 genome (Supplementary Data 1). For MPAO1/P1, these included the essential *ftsY*, an adhesin, several T6SS effectors (see below), and four of the overall eight NRPSs. NRPSs are highly relevant for AMR as they often represent enzymes involved in the biosynthesis of antibiotics^59^. In fact, due to the multi-resistant phenotype of ESKAPE pathogens, concerted efforts aim to describe their NRPS gene clusters in search of new therapeutic approaches^60^, reinforcing the need for complete genome sequences.

Comparative genomics with the PAO1 type strain uncovered an inventory of conserved and strain-specific genes, and a list of genome-wide SNPs, extending an earlier study that had compared a subset of genomic regions^20^. Among the 232 MPAO1-unique gene clusters, bacteriocins^61^ were enriched, which have a role in restricting the growth of closely related microbial competitors to gain an advantage in colonizing a variety of environments^62^. The complete MPAO1 genome enabled us to remap valuable existing Tn-seq data sets from relevant conditions^24^, thereby identifying 39 MPAO1-unique essential genes that had escaped detection so far due to reference-based PAO1 mapping. 18 of these genes were essential in at least 50% of the 16 Tn-seq samples, and six represented general essential genes, including a Phd/YefM family type antitoxin (MPAO1_22380), which was essential in all samples. This is worth noting given the relevance of toxin-antitoxin systems for bacterial growth arrest and persistence^63^. Importantly, our data do not conflict with results from previous studies; rather, they open the field to study the roles of additional MPAO1-unique essential genes. Furthermore, our results suggest that groups planning to construct inventories of core essential genes in other pathogens, following the elegant approach of Poulsen et al.^26^ who had considered both relevant media mimicking different infection types and nine strains from different lineages of a *P. aeruginosa* phylogenetic tree, should ideally select complete genomes without any genomic blind spots.

To leverage the experimental arm of our model (Fig. 7), the consortium developed a PDMS microfluidic flow chamber for biofilm growth, which offers several significant advantages. It provides laminar flow conditions inside the channels (Supplementary Table 4), allows gas exchange, decreases the amount of growth medium, facilitates heat transfer, is inexpensive to replicate, and permits imaging of the biofilm and easy harvesting for biochemical characterization. While the flow chamber can be used to monitor biofilm formation on both glass (oxygen impermeable) and PDMS, it is more relevant to investigate biofilm formation on PDMS as a widely applied biomaterial used in indwelling devices and implants^36^. We observed that biofilms on PDMS formed a more homogeneous layer (Fig. 3b) as compared to the commonly observed mushroom-like structures of *P. aeruginosa* biofilms on glass^64^. This effect is not related to hydrodynamics as a flow chamber that previously has been shown to produce mushroom-like structures^65^ has hydrodynamics (*ū* ≈ 208 µm/s and Re = 0.24) comparable to our microfluidic chip. We speculate that the effect is most likely explained by two differences: (i) PDMS is oxygen permeable and can transport oxygen to the base of the biofilm that then manifests in overall biofilm structure, or (ii) slight differences in media composition.

The microfluidic data from the inter-laboratory trial on strain MPAO1 validated the utility of the flow chamber and allowed us to compare the phenotypes of WT and mutant strains of the UW transposon library. Important genes were identified with a microtiter plate screening assay and subsequently validated with the flow chamber. Proof of principle experiments confirmed the role of *arnB* (PA3552), i.e., a gene relevant for colistin resistance^42,43^, both in biofilms grown in the 96-well plate screen and the flow chamber. In addition, a mutant lacking *cbrB* (PA4726) showed reduced resistance to colistin in biofilm and planktonic cells and formed very low amounts of biofilm in both the microtiter plate and flow chamber. In addition, inactivating *cbrB* was found to be as inhibitory for biofilm formation as inactivating the gene *pslB*, known to negatively influence biofilm matrix synthesis^39^. As part of the two-component system CbrAB, a mutation in the response regulator *cbrB* is known to negatively affect the use of several carbon and nitrogen sources^37^. Such a defect could explain the low growth rate, the low biofilm biomass and therefore the low resistance to colistin of this mutant. Using *P. aeruginosa* PA14, it was shown that a mutation in CbrA improved biofilm formation, while a mutation in CbrB did not^66^. However, these differences might be explained by strains (MPAO1 versus PA14) or growth media used (M9 versus BM2-biofilm medium). In contrast, our screening revealed that inactivating the transhydrogenase *pntAA* induced high biofilm formation, comparable to the known gene *retS*. While redox balance is known to correlate with biofilm morphology^67^, the precise role of *pntAA* remains to be investigated. Together, the combined data of the screen and flow chamber experiments demonstrated that genes previously not implicated in AMR and biofilm formation can be identified and that the function of known genes can be validated.

The differential proteomics data confirmed proteins known to have a role in biofilm formation and growth. These included MuiA, which inhibited swarming motility and enhanced biofilm formation (roles, that were validated in knockout strains)^46^, and CbpD, for which higher protein abundance had been observed in late phases of biofilm growth; accordingly, mutants displayed a lower amount of biofilm growth and exopolysaccharides (EPS)^47^. Similarly, for two other proteins with significantly higher abundance in biofilms, inactivation studies showed that the gene encoding AcnA impaired biofilm formation and was required for microcolony formation^48^, while an increased abundance of PilY1 repressed swarming and increased biofilm formation, as confirmed by knockout experiments^49^. Biofilm exclusive protein expression was observed for MPAO1_00520, the T6SS VgrG1b effector protein^52^, while the adhesin CdrA (MPAO1_24535)^50^ was highly upregulated in biofilms. Both genes were missed in the MPAO1/P1 genome. CdrA forms a two-partner secretion system with CdrB, and both were upregulated under elevated c-di-GMP levels^50^, in line with the upregulation we observed in biofilm. Moreover, an NRPS (MPAO1_14010) and the hypothetical protein MPAO1_19625 were significantly upregulated in biofilm (Table 3). The data provided insights beyond the top differentially abundant proteins. Notable examples included immunity protein TplEi^68^ (PA1509, MPAO1_18250), a bacteriocin of the H2-T6SS^51^, which was exclusively expressed in biofilm (Supplementary Data 1), and upregulation of nine of 14 structural members of H1-T6SS^51^ (Supplementary Fig. 6). Active T6SSs have been associated with chronic infections in cystic fibrosis patients^52^, and H1-T6SS has an important role in the dominance of *P. aeruginosa* in multi-species biofilms^69^. More sensitive and comprehensive proteomics studies are needed to overcome the limitation that only a third of the theoretical proteome was identified with our shotgun proteomics approach, e.g., by combining data-dependent and data-independent acquisition and the use of spectral libraries^70^, allowing a more comprehensive identification of lower abundant and small proteins, or by analyzing additional conditions or mutant strains under which tightly regulated proteins such as the Tse toxins (secreted substrates of the H1-T6SS) are expressed (Supplementary Fig. 6)^71^.

The public MPAO1 (and PAO1) iPtgxDBs allows the identification of missed genes by proteogenomics^31^, which often encode short proteins (sProteins) that can carry out important functions^72,73^. Interestingly, Tn-seq data from the Manoil group had implied an essential genomic region in the PF1 phage region of PAO1-UW^24^. Re-mapping their data, we identified a general essential gene (MPAO1_22380) annotated in our MPAO1 genome whose homolog had been missed in the PAO1 genome annotation, and which appeared to encode the antitoxin member of a ParDE-like TA system (PA0728.1, Fig. 2). However, we did not identify expression evidence for the antitoxin MPAO1_22380 (83 aa) with our iPtgxDB, most likely because our data set (33% of MPAO1 proteins) was not as extensive as that used in a comprehensive proteogenomic study (85% of *Bartonella henselae* proteins)^31^, whose complete membrane proteome coverage included expression evidence for all T4SS members^74^. Nevertheless, we observed proteogenomic evidence for gene products missed in the fragmented MPAO1/P1 genome, for new start sites, and for single amino acid variations, underlining the potential value of proteogenomics for application in clinical proteomics.

Our proof of principle experiments uncovered several candidates for follow-up studies and illustrated the benefit of the complete MPAO1 genome, which led to the discovery of six general essential genes not contained in the transposon library, and which will allow identifying evolutionary changes that lead to AMR in biofilm by deep sequencing in the future. Having been validated for the generation of reproducible inter-laboratory *P. aeruginosa* biofilm results, a milestone en route to a community standard (see Data availability), the microfluidic platform can be instrumental to investigate other biofilms, notably clinical pathogens and mixed-species biofilms^69^. The upregulation of the H1-T6SS highly relevant for dominance of *P. aeruginosa*^69^ implies that our microfluidic chamber should also be valuable for this extension. Our proposed workflow (Fig. 7) with feedback between genotypic and phenotypic assessment of biofilm characteristics can thus be leveraged across the field of biofilm research and helps bridge the gap between genome-wide and reductionist approaches to study phenomena related to biofilm-associated AMR.

## Methods

### Bacterial growth and genomic DNA extraction

*P. aeruginosa* strain MPAO1 (originating from the lab of Dr. Barbara Iglewski) was obtained from Prof. Colin Manoil, UW (Seattle, USA) together with the transposon insertion mutant collection of ~5000 mutated genes (9437 strains)^21^. For DNA extraction, the MPAO1 cryoculture was streaked out on 20% BHI solid medium (7.4 g in 1 L water) containing 1.5% agar (both Sigma, Switzerland). Shaken 20% BHI fluid cultures were inoculated from a single colony and grown at 30 °C until the mid-exponential phase (OD600 = 0.5). Genomic DNA (gDNA) was extracted with the GeneElute kit (Sigma, Switzerland), following the Gram-negative protocol, including RNase treatment. A study that had analyzed 9331 complete bacterial genomes^29^ (NCBI RefSeq, assembly category: ‘complete genome’; status Feb. 23, 2018; see their TableS4) reported that 106 *P. aeruginosa* strains have been sequenced completely, which included only two PAO1 strains (and no complete genome of strain MPAO1). 38/106 (36%) had difficulty assemble genomes with repeat pairs >10 kilo base pairs (bp).

### Sequencing, de novo genome assembly, and annotation

PacBio SMRT sequencing was carried out on an RSII machine (1 SMRT cell, P6-C4 chemistry). A size selection step (BluePippin) was used to enrich for fragments longer than 10 kb. The PacBio run yielded 105,221 subreads (132 Gbp sequence data). Subreads were de novo assembled using the SMRT Analysis portal v5.1.0 and HGAP4^75^, and polished with Arrow. In addition, a 2 × 300 bp paired-end library (Illumina Nextera XT DNA kit) was sequenced on a MiSeq. Polishing of the assembly with Illumina reads, circularization, start alignment using *dnaA,* and final verification of assembly completeness was performed as described previously^76^. The quality of the aligned reads and the final chromosome was assessed using Qualimap^77^. In addition, we checked for any potential large scale mis-assemblies using Sniffles v1.0.8^78^ by mapping the PacBio subreads using NGMLR v0.2.6^78^. SPAdes v3.7.1^79^ was run on the Illumina data to detect smaller plasmids that might have been lost in the size selection step. The genome was annotated with the NCBI’s prokaryotic genome annotation pipeline (v3.3)^80^. Prophages were identified with Phaster^81^. Detailed annotations for all CDS were computed as described previously^82^; this included assignment to Cluster of Orthologous Groups (COG) categories using eggnog-mapper (v1.0.3) and EggNOG 4.5, an Interproscan analysis and prediction or /integration of subcellular localizations, lipoproteins, transmembrane helices and signal peptides (for details, see Supplementary Data 1).

### Comparative genomics of selected PAO1 genomes

The genome of the *P. aeruginosa* PAO1-UW reference strain^2^ was compared to our complete MPAO1 genome using the software Roary (v3.8.0)^83^ to define core and strain-specific gene clusters as described before^30,83^. A BlastP analysis helped to correctly identify conserved genes with ribosomal slippage (*prfB*; *p*eptide chain release factor B) or that encodes selenocysteine (MPAO1_25645), which otherwise can be misclassified as unique genes; genes of 120 bp or below (17 in MPAO1) were not considered. ProgressiveMauve^84^ was used to align the genomes globally and to identify larger genomic differences. Smaller differences (indels, single nucleotide polymorphisms (SNPs)), were identified and annotated against the PAO1 reference strain as described previously^82^. Furthermore, contigs from the MPAO1/P1 genome^32^ were aligned to our complete MPAO1 genome assembly using BWA mem^85^. Bedtools v2.16.1 “genomecov”^86^ was used to calculate a gene-wise coverage, allowing to identify genes that were missed in the 140 contigs.

### Re-mapping of Transposon-sequencing data

MPAO1 Tn-seq data sets^24^ were downloaded from NCBI’s SRA (SRP052838) and mapped back both to the PAO1-UW reference strain genome^2^ and to our MPAO1 assembly following the scripts and notes provided in the Supplement. Insertion sites were computed as described by the authors, reads mapping to multiple genome positions were assigned randomly, and the number of insertion sites per gene was used to differentiate essential and non-essential genes as described^24^. Genes with zero insertions were considered essential; for the remaining genes, normalized read counts across all insertion sites per gene (considering insertions falling within 5–90% of the length of each gene) were log2 transformed and fitted to a normal distribution. Genes with a *p* value < 0.001 were added to the list of essential genes. Finally, essential and conditionally essential genes were identified among the three main growth conditions (LB medium, minimal medium, sputum) as described^24^. Data from each growth condition consisted of multiple mutant pools; for LB, two mutant pools additionally contained multiple replicates (LB-1: 3 replicates; LB-2: 2 replicates). For LB, genes were considered essential in the mutant pool LB-1 and LB-2 if at least two of three (LB-1) and one of two replicates (LB-2) agreed. Next, a consensus set of essential genes in LB and the minimal medium was derived from those genes that were essential in at least two of three mutant pools (LB-1, LB-2, and LB-3) in LB and minimal respectively. Similarly, essential genes in sputum (four mutant pools) were derived if data from at least three of four pools agreed. Finally, genes that were essential in all three growth conditions were called “general essential genes (312)” and genes essential to a specific growth condition were called “condition-specific essential genes”. Together, they comprise “all essential genes (577)”; for further details, see Supplement.

### Microfluidic chamber used for biofilm growth

The standardized microfluidic flow chamber consisted of a PDMS chip with a straight microfluidic channel (30 mm length × 2 mm width × 0.200 mm depth) that naturally adhered to a glass coverslip (26 × 60 mm; thickness no.1). The wafer master was fabricated using SU-8 spin-coated to a thickness of 200 μm on a silicon wafer in advance of standard soft lithography replication into PDMS [84]. From this, polyurethane clones of the structures were prepared to upscale production and for sharing microfluidic molds between laboratories. A degassed 10:1 mixture of Sylgard 184 PDMS base and curing agent was cured in an oven at 60 °C for 2 h. Following cooling and retrieval from the SU-8 wafer the structured PDMS was attached, structures facing upwards, to a silicone baking mold using a transparent double-sided adhesive (3 M). The PDMS part was degassed, while the two-component polyurethane (Smooth-Cast™ 310) solutions were each thoroughly shaken for 10 min and then combined in a 1:1 ratio followed by thorough mixing (by repeat inversion and then shaking). The PDMS device was then submerged in the mixture, with degassing for 10 min, after which the mold was left overnight in a well-ventilated area followed by a hard bake at 60 °C for 4 h. Once cooled the PDMS device was retrieved leaving the polyurethane mold in readiness for replica molding fresh PDMS devices again at 60 °C for 2 h. Importantly, PDMS devices are only retrieved after the polyurethane mold has cooled to room temperature to allow the repeated replication (>100 times) of precision PDMS microfluidic chambers. Inlet and outlet ports were prepared using 1-mm-diameter biopsy punches (Miltex™) and then the device was enclosed using a coverslip that was cleaned with 2% RBS 35 detergent (prepared in demineralized water), rinsed with tap water, then immersed in 96% ethanol and sonicated for 5 min, followed by a final rinse with demineralized water and then autoclaved. The inlet and outlet of the microfluidic flow chamber were connected to a syringe pump with a 25 G needle and waste container, respectively, via sterile PTFE tubing (Smiths Medical, ID 0.38 mm, OD 1.09 mm). The chamber was disinfected by flowing 70% ethanol for 15 min at a rate of 20 μL/min, before rinsing with sterile PBS for 15 min and then flushing with M9 minimal medium (Formedium Ltd, Hunstanto, England) for another 15 min at the same flow rate.

### Device inoculation, biofilm staining, and confocal laser microscopy

*P*. aeruginosa MPAO1 was inoculated with 500 µL of an M9-grown overnight pre-culture and grown for ~16–18 h in 10 mL M9 medium (1x M9 salts supplemented with 2 mM MgSO_4_, 100 μM CaCl_2_ and 5 mM glucose) at 37 °C with gentle rotation (150 rpm) until a cell concentration of 1.5 × 10^9^ bacteria/mL was reached. One mL of the culture was then washed twice with PBS (pH 7.0) by centrifugation at 5000×*g* for 5 min at 10 °C. The bacterial pellet was resuspended and diluted in PBS + 2% M9 such that the final cell suspension contained 3 × 10^8^ bacteria/mL. The microfluidic chamber was set on a hotplate at 37 °C with the glass coverslip in direct contact with the hotplate surface. The freshly prepared bacterial suspension was flown through at a rate of 5 μL/min for 1 h. After 1 h, the bacterial suspension was replaced by M9 medium and run through the system at 5 μL/min for 72 h. After 72 h, CLSM images were taken. The biofilm was stained by flowing 1 mL of Live/Dead (Life Technologies, Oregon, USA) staining solution (1.5 μL Syto9 + 1.5 μL propidium iodide in 1 mL of sterile demineralized water) through the flow chamber at 5 μL/min. Once the channel was filled, the flow was stopped and the biofilm kept in the dark for 30 min to allow dye penetration. Finally, PBS was flown through the system at 5 μL/min for 30 min to remove the staining agent. Confocal imaging was performed using a Leica SP8 with ×63 oil immersion lens (HC PL APO CS2 ×63/1.30, Southampton; LabA), a Leica SP8 with ×63 water immersion lens (HC PL APO ×63/1.20 W CORR CS2; BAM, LabB), and a Leica SP2 with x63 water lens (HCX APO L ×63/0.9 W; Groningen, LabC) for 3 biological repeats comprising 3 technical repeats per site (*n* = 9 biological/*n* = 27 technical). *Z*-stacks (1 μm) were taken of the biofilms formed on the PDMS surface of the device at five separate regions (besides the inlet, 25%, 50%, 75%, and beside the outlet). COMSTAT 2.1 (Image J) analysis of combined confocal data was performed to provide a quantification of average biofilm thickness and Live/Dead biovolume^87^. A 2-way ANOVA multiple comparisons was performed with Tukey’s post hoc test to determine 95% confidence intervals. Similar conditions were applied to strain PA4726 (*cbrB*) that had shown reduced biofilm growth during screening, and PA3552 (*arnB*) which demonstrated robust biofilm formation. Biofilm formation of both mutant strains was compared to the MPAO1 WT strain after 18 h growth in the flow chamber.

### Screening the public MPAO1 transposon library for antibiotic resistance

The protocol to assess the antibiotic resistance of biofilm-forming MPAO1 cells was adapted from a previous study^88^. Frozen mutant stocks of 95 randomly selected mutants of the UW Genome Center’s *P. aeruginosa* PAO1 transposon mutant library^21^, each harboring a transposon insertion inactivating the function of the respective gene, were allowed to recover in 20% BHI overnight at 150 rpm and 37 °C. All subsequent incubations were done at 37 °C in 96-well plates (TPP tissue culture 96-well plates, Z707902, Sigma-Aldrich) covered with an air-permeable foil (Breathe-Easy sealing membrane, Z380059, Sigma-Aldrich) without further shaking. The overnight cultures were diluted 10 fold in M9 medium and 100 µL each was distributed in six plates (1 well/mutant/plate). After 24 h incubation, the biofilm formation from two plates was quantified by crystal violet staining, while biofilms from the other four plates were washed with 0.9% NaCl to remove planktonic bacteria. Bacteria were then exposed to either M9 or M9 supplemented with 25 µg/mL of colistin, i.e., much higher than the minimal inhibitory concentration (MIC) for the planktonic growth of *P. aeruginosa* (4 µg/mL), allowing us to focus specifically on the biofilm bacteria. After 24 h treatment, the medium was removed, biofilms were washed with 0.9% NaCl to remove all traces of antibiotics, and bacteria were allowed to recover in fresh colistin-free M9 medium. After 24 h incubation, the recovery of biofilm bacteria was measured by turbidity (OD600) to reveal if the mutation influences the resistance attributed by the biofilm. To confirm the reliability of our screening, promising mutants were analyzed independently in triplicate. Cell suspensions of each mutant were prepared in M9 medium (5 × 10^6^ CFU/mL) and biofilm biomass was quantified by crystal violet after 24 h incubation at 37 °C. Biofilm cell resistance was quantified by measuring the turbidity of biofilm suspension after 24 h treatment with different concentrations of colistin and after 24 h recovery in fresh M9 medium. Selected mutants of interest were further characterized to assess biofilm formation (as described above), MIC, and MBIC (see Supplementary Fig. 3 for detail).

### Protein extraction from MPAO1 planktonic and biofilm cultures

For planktonic protein extractions, 10 mL MPAO1 was grown overnight (~18 h) in M9 medium under gentle rotation (150 rpm), centrifuged at 4000×*g*/5 min/RT, and the pellet resuspended in 1 mL Hanks’ Balanced Salt Solution (HBSS). Biofilms were grown for 72 h using the microfluidic device as previously described, the PDMS device removed from the glass coverslip, and the combined biofilm biomass from 3 lanes harvested into 1 mL HBSS. Cells from both populations were washed twice in HBSS at 10,000×*g*/5 min/RT and the pellets resuspended in 1 mL lysis buffer (7 M urea, 2 M thiourea, 35 mM CHAPS, 20 mM DTT, 1 M NaCl). Samples were frozen at −80 °C for 30 min and then thawed at 34 °C for 20 min. Trichloroacetic acid (TCA) precipitation was performed by adding the bacterial samples to 100% ice-cold acetone and 100% trichloroacetic acid in a 1:8:1 ratio and precipitating at −20 °C for 1 h. Samples were then centrifuged (18,000×*g*/10 min/4 °C), the supernatant discarded, and the pellet washed twice with 1 mL ice-cold acetone (18,000 × *g*/10 min/4 °C). Acetone was removed, the pellet air-dried at room temperature, and resuspended in 0.1 M Triethylammonium bicarbonate (TEAB) plus 0.1% Rapigest. Protein sample validation was performed by 1DE gel electrophoresis. 19.5 μL sample was added to 7.5 μL NuPAGE LDS buffer and 3 μL NuPAGE reducing reagent, heated at 70 °C for 10 min, then run on a NuPAGE 4–12% Bis-Tris gel with MOPS buffer at 200 V for 50 min alongside a Novex Sharp standard. The gel was stained with SimplyBlue Safe Stain for 1 h, then destained with dH_2_O.

### Protein processing, mass spectrometry, and database search

Protein samples were heated at 80 °C for 10 min, then DTT added at a final concentration of 2 mM and incubated at 60 °C for 45 min. Samples were then briefly vortexed, pulse spun, and cooled to room temperature before adding iodoacetamide to a final concentration of 6 mM. Samples were incubated at room temperature for 45 min (protected from light), vortexed and pulse spun briefly, then trypsin added at a final concentration of 1.3 µg/mL. Following incubation overnight at 37 °C (protected from light), trifluoroacetic acid (TFA) was added to a final concentration of 0.5% then incubated at 37 °C for 30 min. Samples were centrifuged at 13,000×*g* for 10 min at RT, the supernatants removed and vacuum concentrated. The resultant pellets were resuspended in 3% acetonitrile + 0.1% trifluoroacetic acid and peptide quantification performed using the Direct Detect system (Merck Millipore). Protein samples were normalized then vacuum concentrated in preparation for mass spectrometry.

Peptide extracts (1 μg on column) were separated on an Ultimate 3000 RSLC nano system (ThermoScientific) using a PepMap C18 EASY-Spray LC column, 2 μm particle size, 75 μm × 75 cm column (ThermoScientific) over a 140 min (single run) linear gradient of 3–25% buffer B (0.1% formic acid in acetonitrile (v/v)) in buffer A (0.1% formic acid in water (v/v)) at a flow rate of 300 nL/min. Peptides were introduced using an EASY‐Spray source at 2000 V to a Fusion Tribrid Orbitrap mass spectrometer (ThermoScientific). The ion transfer tube temperature was set to 275 °C. Full MS spectra were recorded from 300 to 1500 *m*/*z* in the Orbitrap at 120,000 resolution using TopSpeed mode at a cycle time of 3 s. Peptide ions were isolated using an isolation width of 1.6 amu and trapped at a maximal injection time of 120 ms with an AGC target of 300,000. Higher‐energy collisional dissociation (HCD) fragmentation was induced at an energy setting of 28 for peptides with a charge state of 2–4. Fragments were analyzed in the Orbitrap at 30,000 resolution. Analysis of raw data was performed using Proteome Discoverer software (ThermoScientific) and the data processed to generate reduced charge state and deisotoped precursor and associated product ion peak lists. These peak lists were searched against the *P. aeruginosa* MPAO1 protein database (a max. of one missed cleavage was allowed for tryptic digestion, the variable modification was set to contain oxidation of methionine and N-terminal protein acetylation, and carboxyamidomethylation of cysteine was set as a fixed modification). The FDR was estimated with randomized decoy database searches and was filtered to below 1% FDR at the protein level. Differentially abundant proteins were identified using DESeq2^89^; significantly differentially abundant proteins had an adjusted (multiple testing corrected) *p* value ≤ 0.05 and a log_2_ fold change of ≥ 1 or ≤−1.

### Proteogenomics

An iPtgxDB was created for *P. aeruginosa* MPAO1 as described previously^31^, using the NCBI annotation as anchor annotation. Ab initio gene predictions from Prodigal^90^ and ChemGenome^91^ and a modified in silico prediction that considers alternative start codons (TTG, GTG, CTG) and ORFs above 6 amino acids (aa) in length were integrated into a step-wise fashion. Proteomics data from MPAO1 cells grown planktonically or as biofilm were searched against this iPtgxDB with MS-GF+ (v2019.04.18)^92^ using Cysteine carbamidomethylation as fixed, and oxidation of methionine as variable modifications. Using the target-decoy approach of MS-GF+, the FDR at the PSM level was estimated and filtered below 0.2%. Only unambiguous peptides as identified by a PeptideClassifier analysis^55^, using the extension that supports proteogenomics for prokaryotes^31^, were considered.

### Reporting summary

Further information on research design is available in the Nature Research Reporting Summary linked to this article.

## Supplementary information

Supplementary Information

Dataset1

Dataset 2

Dataset 3

Dataset 4

Dataset 5

Reporting Summary Checklist

## Data Availability

The MPAO1 genome sequence is available at NCBI Genbank (acc# CP027857; Bioproject: PRJNA438597, Biosample: SAMN08722738). Read data are available under SRR10153205 (Illumina) and SRR10153206 (PacBio). Proteomics data are available from PRIDE (acc# PXD017122) upon acceptance of the manuscript. The iPtgxDBs for *P. aeruginosa* MPAO1 and PAO1 are available from https://iptgxdb.expasy.org, both as a searchable protein database (FASTA format) and a GFF file, which can be loaded in a genome viewer and overlaid with experimental evidence. Biofilm growth data from the microfluidic chamber will be made available at 10.21253/DMU.c.4851483. To support technology dissemination, the polyurethane master molds of the microfluidic chambers are available upon request from the UoS/NBIC; a CAD file can be found as Supplementary Data 5. Code availability: all analyses presented rely on open source software or published code that is referenced.

## Code availability

Open-source R code used for genomic, Tn-seq, and proteomics data processing is available from the authors upon request.
