# Supplementary material for: An integrated model system to gain mechanistic insights into biofilm-associated antimicrobial resistance in Pseudomonas aeruginosa MPAO1
Source: NPJ Biofilms Microbiomes. 2020 Oct 30;6:46. doi: 10.1038/s41522-020-00154-8 (PMC7603352; doi:10.1038/s41522-020-00154-8)
Supplement: Supplementary file 1 — Supplementary Information [file 41522_2020_154_MOESM1_ESM.pdf]

Supplemental Material

Table of Contents

Supplementary Table 1. Genome characteristics of *P. aeruginosa* MPAO1 and PAO1-UW. ... 2

Supplementary Data 1. Detailed annotation & integrated information for data mining (Master table). ... 3

Supplementary Figure 1. The genome of strain MPAO1/P1 contains more interrupted genes. 4

Supplementary Data 2. Summary of SNP differences between strain MPAO1 and PAO1. ... 5

Supplementary Data 3. List of shared and specific gene clusters for strains PAO1 and MPAO1. ... 5

Supplementary Table 2. Gene ontology categories among 232 unique MPAO1 gene clusters. ... 6

Supplementary Table 3. Re-mapping of Tn-seq data against the MPAO1 genome leads to a higher percentage of mapped reads. ... 7

Supplementary Figure 2. Overview of conditionally essential genes in *P. aeruginosa* MPAO1. ... 8

Supplementary Data 4. Summary table of 1117 genes essential in at least one Tn-seq library. ... 8

Supplementary Table 4. Laminar flow conditions achieved in the biofilm chamber. ... 9

Supplementary Table 5. List of *P. aeruginosa* MPAO1 mutant strains used in this work. ... 10

Supplementary Figure 3. Diagram of the screening protocol to measure biofilm formation and biofilm cell resistance towards colistin. ... 13

Supplementary Figure 4. Growth kinetics of WT MPAO1 and mutant strains in 96 well plates. ... 14

Supplementary Figure 5. Rank of estimated protein abundances in planktonic and biofilm cells. ... 15

Supplementary Figure 6. Several members of the H1-T6SS are upregulated in biofilm. ... 17

Supplementary Table 6. Summary table of annotation clusters created for the MPAO1 & PAO1 iPtgxDBs. ... 18

Supplementary Data 5. Design of the microfluidic chamber as a CAD file. ... 18

References ... 19

Supplementary Table 1. Genome characteristics of *P. aeruginosa* MPAO1 and PAO1-UW.

	***P. aeruginosa* MPAO1**	***P. aeruginosa* PAO1-UW***
Genbank accession #	CP027857	NC_002516
No. chromosomes (plasmids)	1 (0)	1 (0)
Size (bp)	6,275,467	6,264,404
G+C content (%)	66.5	66.6
Coverage (PacBio)	180x	n.a.
Coverage (Illumina MiSeq)	101x	n.a.
Total No. of genes	5,926	5,697
No. of protein-coding genes (CDSs)	5,799	5,572
No. of rRNA operons (16S, 23S, 5S)	4,4,4	4,4,4
No. of tRNA genes	63	63
No. of pseudogenes	48	19
No. of ncRNA, tmRNA	4, -	29, 1
No. of 4.5S rRNA	-	1
Prophages	3	2

*We interchangeably use *P. aeruginosa* PAO1 or PAO1-UW.

Supplementary Data 1. Detailed annotation & integrated information for data mining (Master table).

See separate Excel file. The file contains detailed annotation for all 5,799 annotated MPAO1 CDS. Furthermore, it includes information whether the genes are conserved or unique compared to *P. aeruginosa* PAO1, the respective PAO1 homolog (and gene name, where applicable), the genes missed in three Illumina short read-based assemblies of MPAO1 strains [1] [2], gene essentiality status from our study and previous data sets [3] [4], protein expression evidence from our study (biofilm versus planktonic growth), and additional information about protein domains, families, patterns, signatures, a Gene Ontology (GO) classification, a prediction of subcellular localization, lipoproteins, etc. We did not specifically assess short MPAO1-unique genes, whose gene essentiality status is more difficult to robustly classify. Instead, we added a proteogenomics element to enable identification of novel short proteins (see main article).

Supplementary Figure 1. The genome of strain MPAO1/P1 contains more interrupted genes.

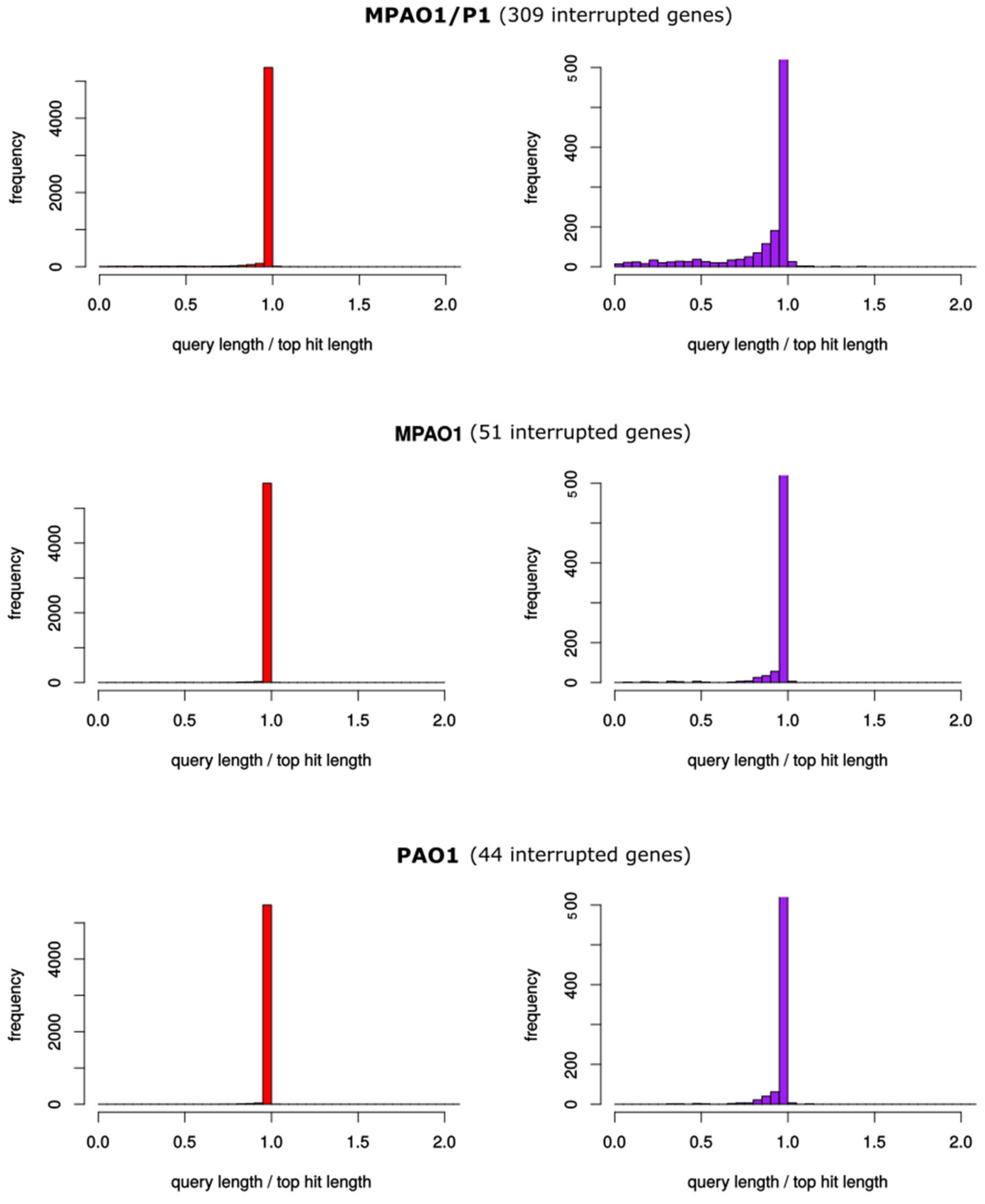

An analysis with Ideel (https://github.com/mw55309/ideel) uncovered large differences of the number of predicted pseudogenes/interrupted genes, which can serve as one parameter to estimate genome completeness. For MPAO1/P1 (5,791 CDS), about six times as many putative pseudogenes/interrupted genes were identified compared to the complete MPAO1 (5,799 CDS) and PAO1-UW (5,572 CDS) genome sequences (MPAO1/P1: 309; MPAO1: 51; PAO1-UW: 44). A complete genome typically shows a narrow peak around 1, i.e., most of the CDS have a full length BlastP hit against the respective UniProt entry, and a shallow tail of the distribution towards the left (see zoomed region in the right plots).

Supplementary Data 2. Summary of SNP differences between strain MPAO1 and PAO1.

The table (see separate Excel file) lists the differences between our complete genome assembly of MPAO1 and the genome sequence of PAO1-UW [5], the PAO1 type strain. We could confirm the 16 SNPs reported previously for both strains [6], one SNP only in MPAO1 and six SNPs observed as base exchanges in both strains. We could also confirm nine of the SNPs that had been reported for the PAO1 DSM strain only [6] (synonymous substitutions in Phage pf1 protein) which are located at the beginning of the inversion region, one SNP in transcriptional regulator MexT and one intergenic SNP at position 5,033,102 of the MPAO1 genome. In addition, we observed a total of 176 additional SNPs and INDELs between PAO1 and MPAO1 that were not reported by Klockgether and colleagues, as their comparison had focused on selected genomic regions of the two strains [6].

Supplementary Data 3. List of shared and specific gene clusters for strains PAO1 and MPAO1.

Gene clusters specific to either PAO1 (21) or MPAO1 (232), and those shared between the two strains (5,534) as returned from an analysis with Roary [7] are listed in a separate Excel file, along with genomic coordinates, annotation and COG classification. For the MPAO1-specific gene clusters, information about essentiality was computed based on the very important dataset by Lee and colleagues and using the scripts they provided in their Supplementary Material [3].

Supplementary Table 2. Gene ontology categories among 232 unique MPAO1 gene clusters.

GO accession	GO description	Unique genes	total proteins annot.	unique proteins annot.	p-value
**Biological Process**					
GO:0006468	protein phosphorylation	MPAO1_11765, MPAO1_24875, MPAO1_24880	10	3	2.90E-05
GO:0030153	bacteriocin immunity	MPAO1_05695, MPAO1_20105	5	2	0.00041
GO:0006571	tyrosine biosynthetic process	MPAO1_09400	1	1	0.00664
GO:0006470	protein dephosphorylation	MPAO1_24870	5	1	0.03276
GO:0015074	DNA integration	MPAO1_24800	7	1	0.04557
**Molecular Function**					
GO:0004672	protein kinase activity	MPAO1_11765, MPAO1_24875, MPAO1_24880	85	3	0.0226
GO:0003866	3-phosphoshikimate 1-carboxyvinyltransferase	MPAO1_09400	1	1	0.0073
GO:0004308	exo-alpha-sialidase activity	MPAO1_11350	1	1	0.0073
GO:0004665	prephenate dehydrogenase (NADP+) activity	MPAO1_09400	1	1	0.0073
GO:0008849	enterochelin esterase activity	MPAO1_13245	1	1	0.0073
GO:0008977	prephenate dehydrogenase (NAD+) activity	MPAO1_09400	1	1	0.0073
GO:0008998	ribonucleoside-triphosphate reductase activity	MPAO1_16050	2	1	0.0145
GO:0004722	protein serine/threonine phosphatase activity	MPAO1_24870	3	1	0.0217
GO:0015643	toxic substance binding	MPAO1_20105	3	1	0.0217
GO:0005102	receptor binding	MPAO1_24810	4	1	0.0288
GO:0016805	dipeptidase activity	MPAO1_06140	4	1	0.0288

GO categories for which only one gene among the unique MPAO1 genes was affected are shown in gray

Supplementary Table 3. **Re-mapping of Tn-seq data against the MPAO1 genome leads to a higher percentage of mapped reads.**

Library Name	# mapped reads		% increase mapped reads	# unique insertion sites		% increase unique insertion sites
	PAO1-UW	MPAO1		PAO1-UW	MPAO1	
LB-1_Rep1	2,095,460	2,099,090	0.17%	92,731	92,938	0.22%
LB-1_Rep2	13,409,799	13,427,972	0.14%	89,678	89,884	0.23%
LB-1_Rep3	8,970,876	8,990,866	0.22%	64,155	64,298	0.22%
LB-2_Rep1	14,495,458	14,546,760	0.35%	82,060	82,239	0.22%
LB-2_Rep2	18,630,794	18,648,695	0.10%	82,441	82,655	0.26%
LB_3	23,922,898	23,952,863	0.13%	110,102	110,331	0.21%
Minimal-1	27,740,278	27,770,384	0.11%	193,648	194,051	0.21%
Minimal-2	13,365,514	13,383,055	0.13%	204,750	205,193	0.22%
Minimal-3	6,441,754	6,449,398	0.12%	250,831	251,570	0.29%
Sputum-1	5,630,125	5,635,321	0.09%	78,706	78,921	0.27%
Sputum-2	7,609,070	7,617,184	0.11%	181,365	181,737	0.21%
Sputum-3	14,491,394	14,508,287	0.12%	48,809	48,930	0.25%
Sputum-4	2,991,136	2,994,255	0.10%	91,856	92,035	0.19%
HumanSerum	754,467	755,143	0.09%	127,543	127,808	0.21%
0.1XLB	8,735,565	8,743,902	0.10%	121,430	121,679	0.21%
BHI	4,438,845	4,443,736	0.11%	174,419	174,796	0.22%

As expected, consistently higher percentages of mapped reads were achieved when mapping the Tn-seq datasets to the complete genome assembly of *P. aeruginosa* strain MPAO1 compared to mapping it to the reference strain PAO1-UW.

Supplementary Figure 2. Overview of conditionally essential genes in *P. aeruginosa* MPAO1.

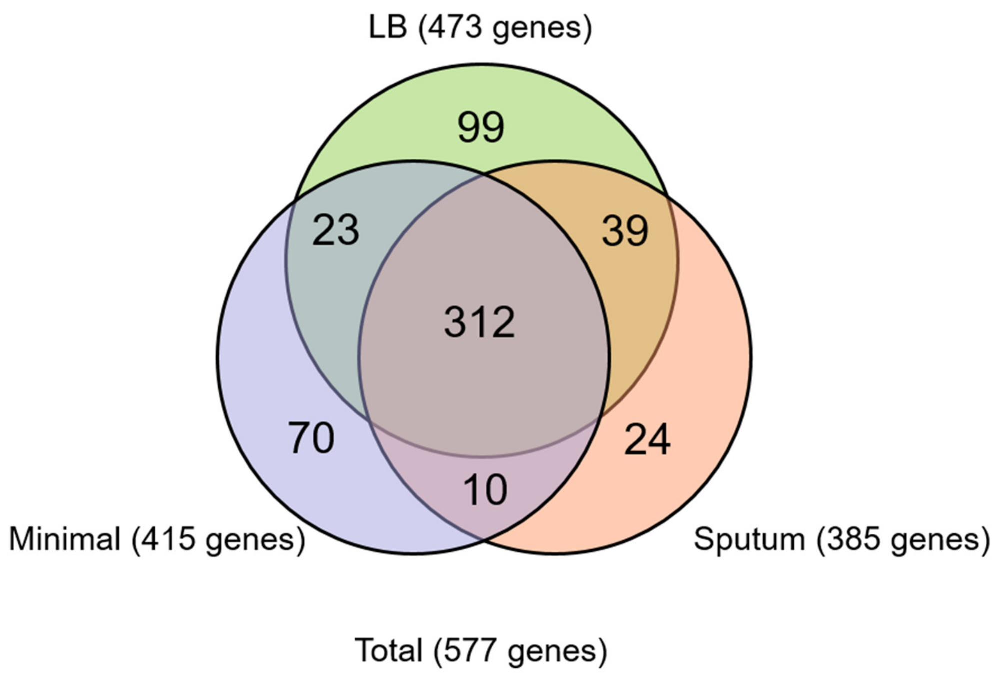

Using the scripts released by Lee and colleagues [3], Tn-seq data were re-mapped to our complete MPAO1 genome and compared with data published, i.e., the set of genes essential in either one of the three conditions sputum, minimal medium and LB medium (577 "all essential genes"), as well as the 312 genes essential in all 3 categories ("general essential genes"). This analysis was close to the original results. Due to the higher mapping success of reads to the complete genome sequence, we identified 39 MPAO1-unique "all essential genes" in the MPAO1 genome (Table 2 and Table S5). Overall, 1117 genes were identified as essential in at least one Tn-seq library; thereof, 136 were MPAO1-unique genes (see Table S7). Six of the MPAO1-unique genes were general essential genes.

Supplementary Data 4. Summary table of 1117 genes essential in at least one Tn-seq library.

See separate Excel file. For completeness, we also show the MPAO1-unique genes that were identified as essential in at least one of the sixteen Tn-seq samples (136; Table S7). The second column shows in how many of the 16 samples a respective gene was called essential. The last three columns indicate the subset of 577 genes essential in at least one of the three primary growth conditions (LB, minimal and sputum), and the subset of 312 genes essential in all three primary growth conditions, i.e., general essential genes (see Methods).

Supplementary Table 4. Laminar flow conditions achieved in the biofilm chamber.

The flow in the flow chamber had a defined laminar flow. The calculated Reynolds Number (see formulas below table) was 0.103 given a volumetric flow rate 5 µL/min, pressure 0.0108 mbar, the channel specifications given below, and correcting for the viscosity and density of water at 37 °C.

Initial Conditions	
Channel geometry	rectangular
Channel height	200 µm
Channel width	2000 µm
Channel length	30 mm
Hydraulic diameter (D)	363.64 µm
Fluid viscosity (µ)	0.73 cP (Pa.s / 10^3)
Fluid density (d)	0.993 g/cm^3 (kg/m^3 x10^3)
**Results**	
Pressure	0.0107652 mbar
Flow rate (Q)	5 µL/min
Flow velocity (v)	0.000208333 m/s
Reynolds number (Re)	0.103051 (**laminar flow**)

$$Re = \frac{d * D * v}{\mu}$$

$$D = \frac{4A}{P}$$

A: section area

P: wetted perimeter

$$v = \frac{Q}{A}$$

Supplementary Table 5. List of *P. aeruginosa* MPAO1 mutant strains used in this work.

All mutants are from the UW Genome Center *P. aeruginosa* MPAO1 transposon mutant library (laboratory of Prof. Dr. Colin Manoil). Listed are: the name of the mutant strain, the identifier of the respective gene in PAO1-UW, the MPAO1 locus tag, the gene name and a putative function. Note: gene names in the UW library are listed as PA0160-G03::ISphoA/hah (given here for strain PW1274 as an example). Most clones are from the 96-well plate with the *arnB* mutant strain (PW7021, PA3552); several additional strains used as positive/negative controls for biofilm formation and to validate selected genes of interest are shown in dark blue.

Strain name	PAO1 gene identifier	MPAO1 locus tag	Gene name	Putative function
PW1156	**PA0095**	MPAO1_00520	*vgrG1b*	conserved hypothetical protein
PW1274	**PA0160**	MPAO1_00860		hypothetical protein
PW9283	**PA0195**	MPAO1_01040	*pntAA*	putative NAD(P) transhydrogenase, subunit alpha part 1
PW1536	**PA0292**	MPAO1_01545	*aguA*	agmatine deiminase
PW1635	**PA0344**	MPAO1_01825		hypothetical protein
PW7893	**PA0357**	MPAO1_01890	*mutM*	formamidopyrimidine-DNA glycosylase
PW1722	**PA0391**	MPAO1_02065		hypothetical protein
PW1808	**PA0440**	MPAO1_02325		probable oxidoreductase
PW1871	**PA0476**	MPAO1_02520		probable permease
PW2290	**PA0711**	MPAO1_22480		hypothetical protein
PW2385	**PA0761**	MPAO1_22195	*nadB*	L-aspartate oxidase
PW2642	**PA0898**	MPAO1_21485	*aruD*	succinylglutamate 5-semialdehyde dehydrogenase
PW2661	**PA0914**	MPAO1_21390		hypothetical protein
PW3188	**PA1211**	MPAO1_19795		hypothetical protein
PW3211	**PA1224**	MPAO1_19730		probable NAD(P)H dehydrogenase
PW3242	**PA1245**	MPAO1_19625	*aprX*	hypothetical protein [8]
PW3395	**PA1320**	MPAO1_19225	*cyoD*	cytochrome o ubiquinol oxidase subunit IV
PW3497	**PA1373**	MPAO1_18945	*fabF2*	3-oxoacyl-acyl carrier protein synthase II
PW3499	**PA1374**	MPAO1_18940		hypothetical protein
PW3660	**PA1467**	MPAO1_18470		hypothetical protein
PW3679	**PA1486**	MPAO1_18370		hypothetical protein
PW3738	**PA1518**	MPAO1_18205		conserved hypothetical protein
PW3859	**PA1599**	MPAO1_17765		probable transcriptional regulator
PW3904	**PA1629**	MPAO1_17615		probable enoyl-CoA hydratase/isomerase
PW4005	**PA1693**	MPAO1_17275	*pscR*	translocation protein in type III secretion

PW4095	**PA1755**	MPAO1_16955		hypothetical protein
PW4171	**PA1804**	MPAO1_16665	*hupB*	DNA-binding protein HU
PW4255	**PA1855**	MPAO1_16390		hypothetical protein
PW4329	**PA1900**	MPAO1_16160	*phzB2*	probable phenazine biosynthesis protein
PW4474	**PA1997**	MPAO1_15660		probable AMP-binding enzyme
PW4590	**PA2084**	MPAO1_15180		probable asparagine synthetase
PW4774	**PA2216**	MPAO1_14460		conserved hypothetical protein
PW4798	**PA2232**	MAPO1_14370	*pslB*	probable phosphomannose isomerase/GDP-mannose
PW4975	**PA2361**	MPAO1_13710		hypothetical protein
PW5204	**PA2510**	MPAO1_12945	*catR*	transcriptional regulator CatR
PW5263	**PA2542**	MPAO1_12765		conserved hypothetical protein
PW5552	**PA2716**	MPAO1_11820		probable FMN oxidoreductase
PW5647	**PA2774**	MPAO1_11460		hypothetical protein
PW5732	**PA2825**	MPAO1_11185	*ospR*	probable transcriptional regulator
PW5757	**PA2838**	MPAO1_11120		probable transcriptional regulator
PW5923	**PA2928**	MPAO1_10660		hypothetical protein
PW6078	**PA3029**	MPAO1_10130	*moaB2*	molybdopterin biosynthetic protein B2
PW6080	**PA3030**	MPAO1_10125	*mobA*	molybdopterin-guanine dinucleotide biosynthesis protein
PW6141	**PA3064**	MPAO1_09940	*pelA*	PelA
PW6275	**PA3137**	MPAO1_09535		probable major facilitator superfamily (MFS) transporter
PW6504	**PA3279**	MPAO1_08785	*oprP*	Phosphate-specific outer membrane porin OprP precursor
PW6554	**PA3305**	MPAO1_08630		hypothetical protein
PW6689	**PA3374**	MPAO1_08270		conserved hypothetical protein
PW6719	**PA3391**	MPAO1_08185	*nosR*	regulatory protein NosR
PW6755	**PA3410**	MPAO1_08085		probable sigma-70 factor, ECF subfamily
PW6868	**PA3470**	MPAO1_07770		hypothetical protein
PW6935	**PA3507**	MPAO1_07575		probable short-chain dehydrogenase
PW6985	**PA3534**	MPAO1_07440		probable oxidoreductase
PW7021	**PA3552**	MPAO1_07345	*arnB*	ArnB
PW7067	**PA3574**	MPAO1_07230	*nalD*	probable transcriptional regulator
PW7125	**PA3606**	MPAO1_07045		conserved hypothetical protein
PW7145	**PA3618**	MPAO1_06985		conserved hypothetical protein
PW7383	**PA3772**	MPAO1_06190		hypothetical protein
PW7530	**PA3870**	MPAO1_05670	*moaA1*	molybdopterin biosynthetic protein A1
PW7566	**PA3890**	MPAO1_05560		probable permease of ABC transporter
PW7664	**PA3939**	MPAO1_05300		hypothetical protein
PW7797	**PA4018**	MPAO1_04895		hypothetical protein
PW8020	**PA4143**	MPAO1_04250		probable toxin transporter
PW8169	**PA4224**	MPAO1_03835	*pchG*	pyochelin biosynthetic protein PchG
PW8212	**PA4282**	MPAO1_22735		probable exonuclease

PW8258	**PA4305**	MPAO1_22850	*rcpC*	RcpC
PW8322	**PA4338**	MPAO1_23025		hypothetical protein
PW8365	**PA4362**	MPAO1_23150		hypothetical protein
PW8394	**PA4378**	MPAO1_23230	*inaA*	InaA protein
PW8466	**PA4434**	MPAO1_23525		probable oxidoreductase
PW8707	**PA4578**	MPAO1_24275		hypothetical protein
PW8795	**PA4625**	MPAO1_24535	*cdrA*	hypothetical protein [9]
PW8936	**PA4711**	MPAO1_25105		hypothetical protein
PW8957	**PA4721**	MPAO1_25155		conserved hypothetical protein
PW8965	**PA4726**	MPAO1_25185	*cbrB*	two-component response regulator CbrB
PW9094	**PA4812**	MPAO1_25645	*fdnG*	formate dehydrogenase-O, major subunit
PW9164	**PA4856**	MPAO1_25880	*retS*	regulator of Exopolysaccharide and Type III Secretion
PW9226	**PA4888**	MPAO1_26040	*desB*	acyl-CoA delta-9-desaturase, DesB
PW9246	**PA4899**	MPAO1_26095		probable aldehyde dehydrogenase
PW9431	**PA5020**	MPAO1_26730		probable acyl-CoA dehydrogenase
PW9494	**PA5058**	MPAO1_26935	*phaC2*	poly(3-hydroxyalkanoic acid) synthase 2
PW9509	**PA5073**	MPAO1_27010		hypothetical protein
PW9793	**PA5219**	MPAO1_27780		hypothetical protein
PW9815	**PA5234**	MPAO1_27860		probable oxidoreductase
PW9856	**PA5261**	MPAO1_28000	*algR*	alginate biosynthesis regulatory protein AlgR
PW9891	**PA5281**	MPAO1_28105		probable hydrolase
PW9895	**PA5283**	MPAO1_28115		probable transcriptional regulator
PW9934	**PA5304**	MPAO1_28225	*dadA*	D-amino acid dehydrogenase, small subunit
PW9970	**PA5324**	MPAO1_28325		probable transcriptional regulator
PW10024	**PA5355**	MPAO1_28485	*glcD*	glycolate oxidase subunit GlcD
PW10082	**PA5384**	MPAO1_28660		probable lipolytic enzyme
PW10153	**PA5423**	MPAO1_28855		hypothetical protein
PW10195	**PA5442**	MPAO1_28970		conserved hypothetical protein
PW10219	**PA5455**	MPAO1_29035		hypothetical protein
PW10275	**PA5484**	MPAO1_29190		probable two-component sensor

Supplementary Figure 3. Diagram of the screening protocol to measure biofilm formation and biofilm cell resistance towards colistin.

The assay is performed in 96-well plates in which biofilms of *P. aeruginosa* MPAO1 are observed to grow on the wall of the well at the interface between air/liquid. A side-view of the well is represented in the diagram below to show the behavior of biofilms formed by a resistant strain (MPAO1 WT) and a sensitive one (*arnB* mutant). Biofilm formation is quantified by crystal violet staining after 24h incubation in M9 medium. Biofilm resistance is evaluated by measuring the ability of treated biofilm cells to recover in antibiotic-free M9 medium. The protocol was adapted from [10]. The minimal inhibitory concentration (MIC) was determined using the same bacterial preparation incubated in M9 supplemented with a gradient of colistin concentrations (from 0.25 to 16 µg/mL). Turbidity was measured after 24h treatment; the MIC was determined as the lowest colistin concentration needed to achieve 90% reduction compared to the turbidity of the untreated condition. The minimal biofilm inhibitory concentration (MBIC) was determined by measuring the recovery of biofilm cells after 24h treatment in M9 medium supplemented with a gradient of colistin concentrations (from 4 to 200 µg/mL). MBIC 50 and MBIC 90 were determined as the lowest concentration of colistin needed to achieve 50 and 90% reduction of biofilm recovery compared to untreated ones, respectively.

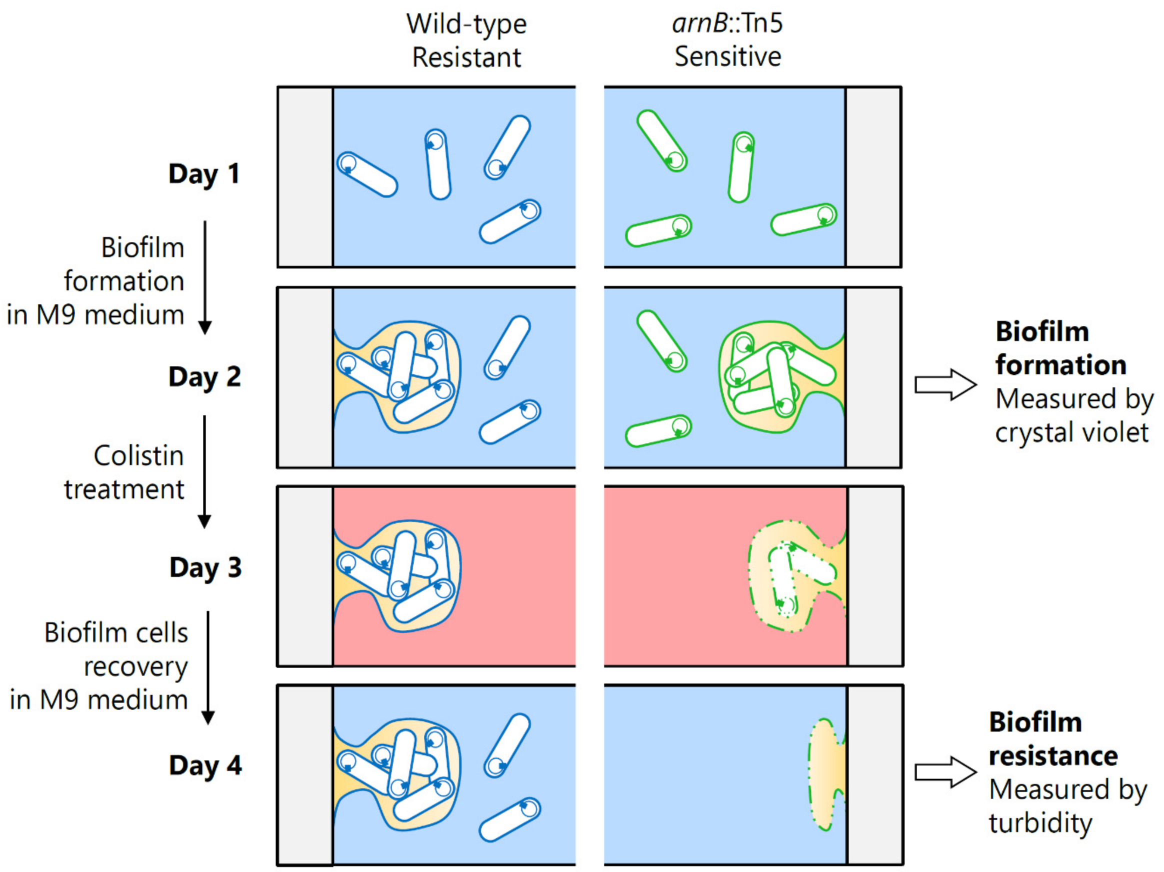

Supplementary Figure 4. Growth kinetics of WT MPAO1 and mutant strains in 96 well plates.

The growth kinetics of the *cbrB* mutant strain (PA4726; blue triangles) is showing a delayed exponential growth in comparison to the WT and to the other transposon mutants when grown in the 96 well plate. Results represent the mean ± standard deviation of one biological experiment (two for WT, *arnB* and *cbrB* mutants) with five technical replicates each.

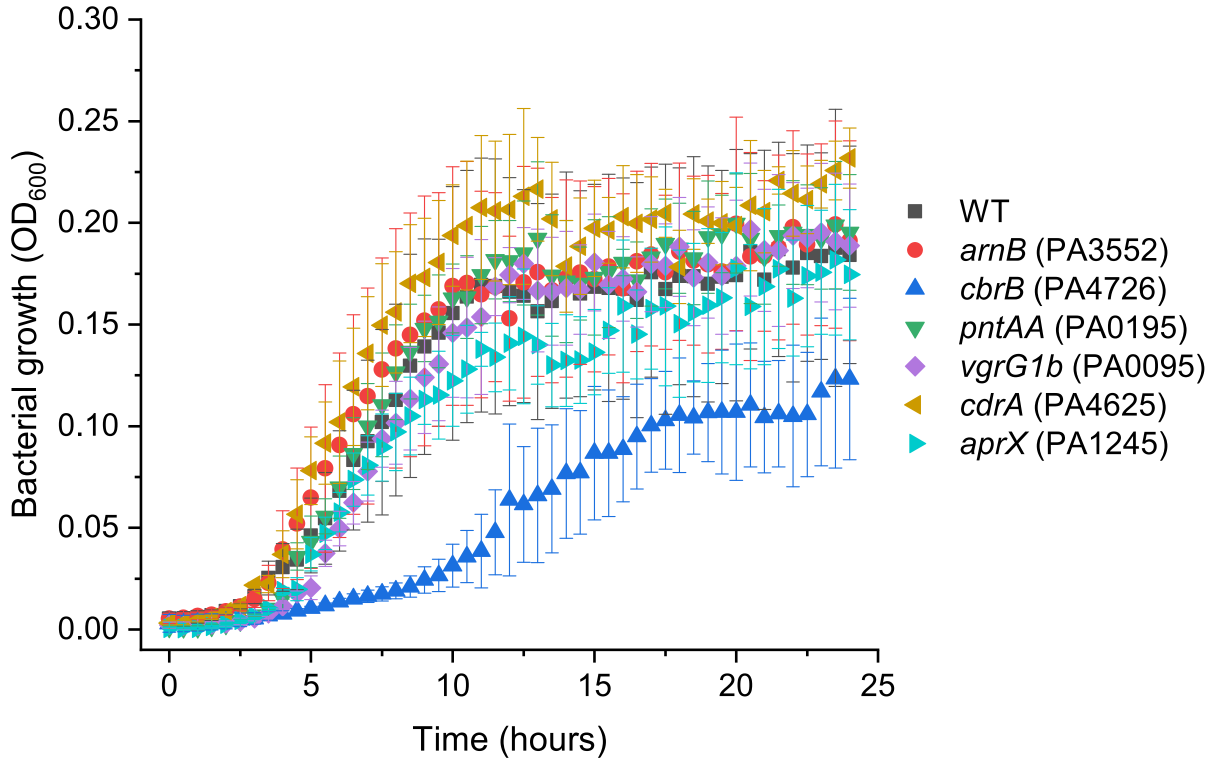

Supplementary Figure 5. Rank of estimated protein abundances in planktonic and biofilm cells.

We calculated the abundance of all proteins that were expressed under planktonic growth conditions (3A) and in the bioifilm flowcell (3B). The respective rank of three top differentially abundant proteins is shown in color (MPAO1_00520, red; MPAO1_19625, green; MPAO1_24535, blue). These proteins are not expressed in planktonic cells (below the threshold), but highly abundant in biofilm cells, with ranks 159, 359 and 956, respectively, and in each replicate (see Table below).

Protein	Total # Pept. planctonic	Total # PSMs planctonic	**PSM repl. 1**	**PSM repl. 2**	**PSM repl. 3**	Total # Pept. biofilm	Total # PSMs biofilm	**PSM repl. 1**	**PSM repl. 2**	**PSM repl. 3**	Log 2 FC	padj
MPAO1_ 00520	0	0	**0**	**0**	**0**	21	21	**7**	**9**	**5**	5.41	0.014
MPAO1_ 19625	1	1	**0**	**0**	**1**	27	43	**11**	**16**	**15**	5.45	0.003
MPAO1_ 24535	1	1	**0**	**0**	**1**	79	89	**23**	**34**	**32**	6.54	0.000

A.

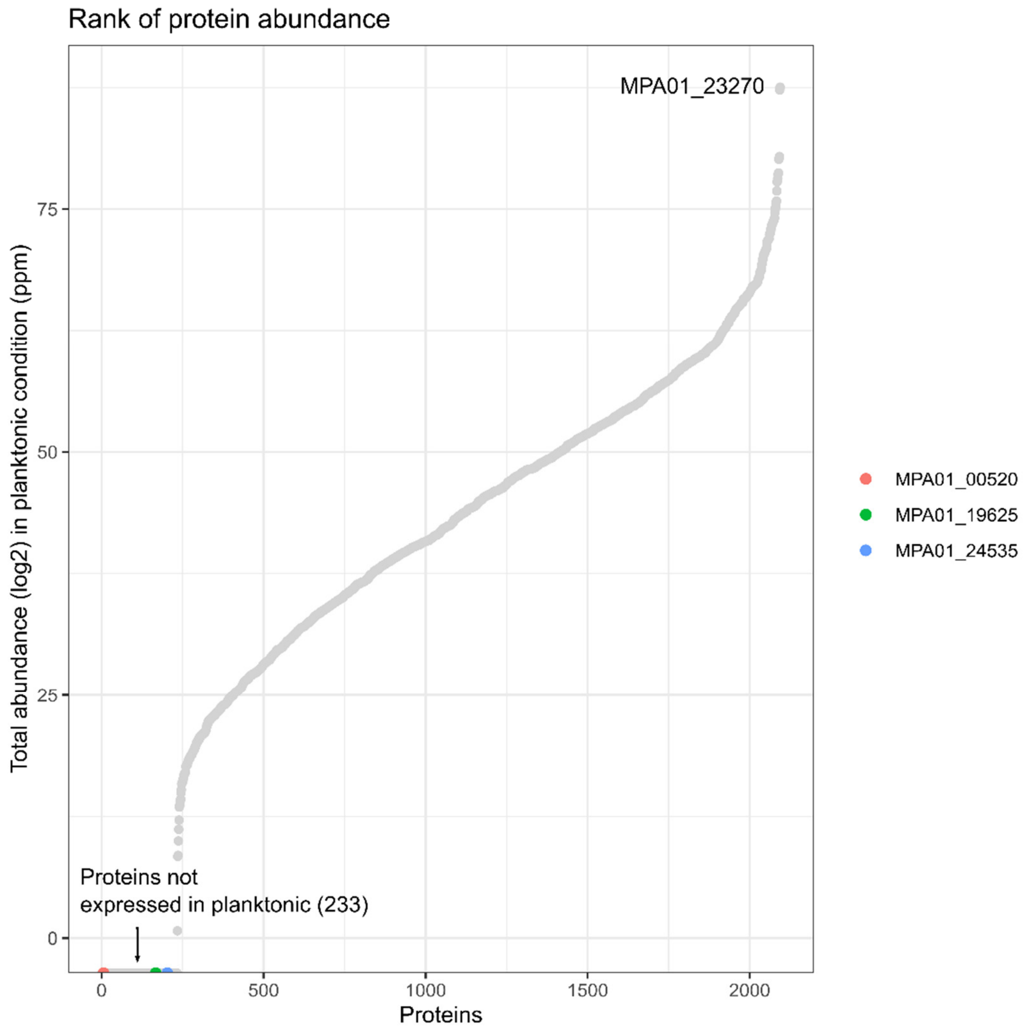

B.

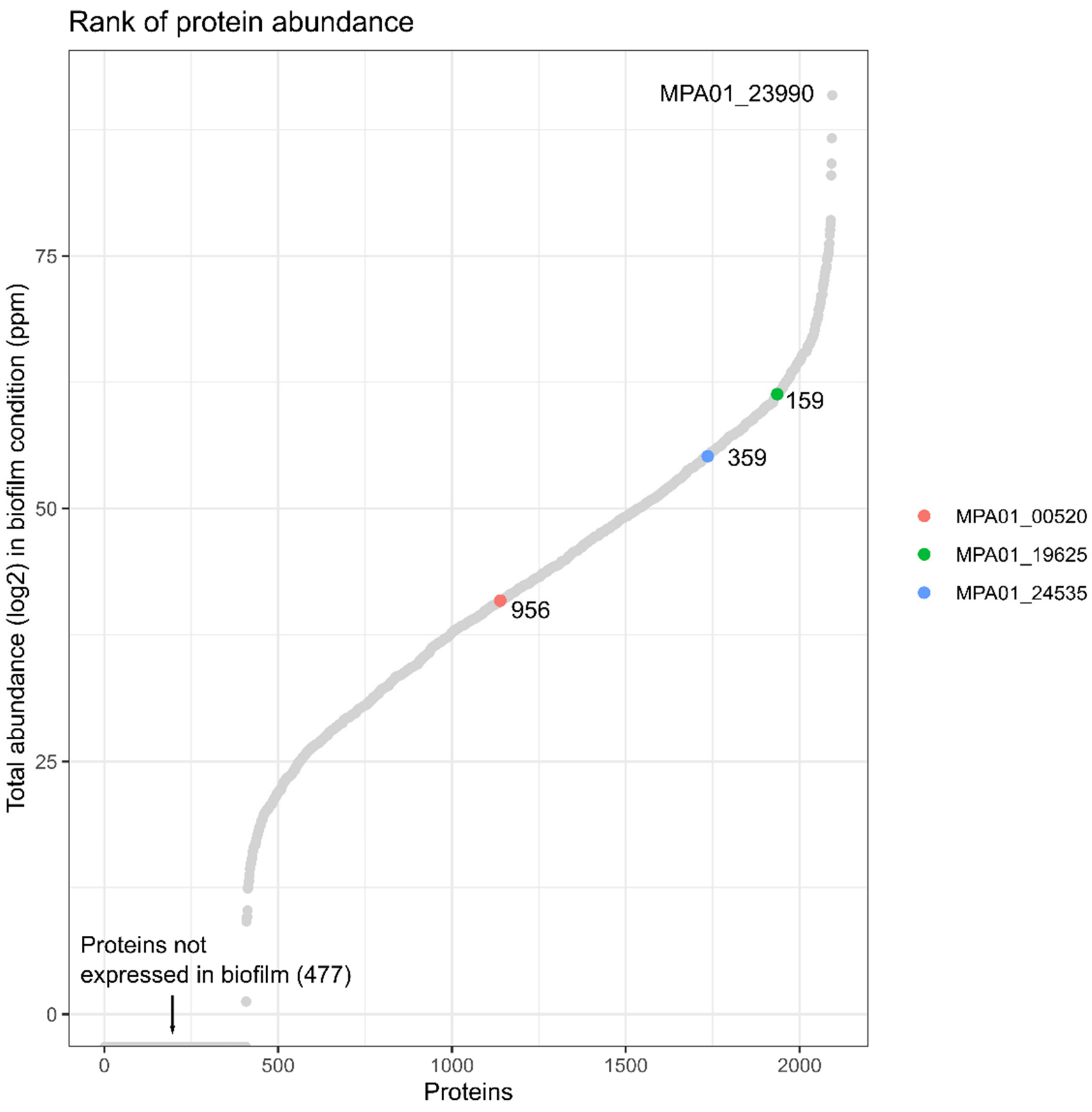

Importantly, all three proteins were identified as expressed in other studies [11-14], two of the PAO1 orthologs are listed as expressed in the Pseudomonas genome database, and protein expression evidence was reported for MPAO1_19625, also called AprX [8], and MPA01_24535, also called CdrA [9].

	Lampaki et al [11]	*Herbst et al [12]*	*Toyofuku et al [13]*	*Kumari et al [14]*
	PAO1	*MPAO1*	*PAO1*	*PAO1*
MPAO1_00520 (PA0095), Vgr1b	-	***yes***	*no*	***yes***
MPAO1_19625 (PA1245), AprX	-	***yes***	*no*	*no*
MPAO1_24535 (PA4625), CdrA	***yes***	***yes***	***yes***	***yes***

Supplementary Figure 6. **Several members of the H1-T6SS are upregulated in biofilm.**

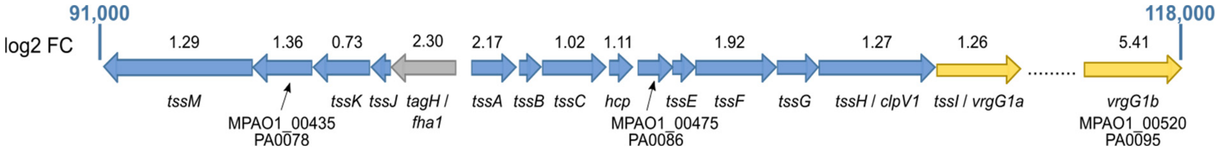

Genomic region of *P. aeruginosa* MPAO1 that shows part of the H1-T6SS region (roughly from nucleotide 91,000-118,000); gene names or MPAO1 locus tags are shown (with the respective PAO1 homolog below). The colors were selected as in 12 and indicate structural elements (blue), vgrGs (yellow) and other known T6SS genes (gray). The shotgun proteomics data (log2 fold change biofilm over planktonic is shown above the arrows) indicated that several (9 of 14, 64%) of the proteins encoded by structural elements of the H1-T6SS and secreted proteins [15] were upregulated in biofilm cells compared to planktonic cells. The H1-T6SS has been described as "a molecular gun firing toxins (Tse1-Tse7)" and has been implied "to challenge the survival of other bacteria and help *P. aeruginosa* prevail in specific niches" [16]. This specific H1-T6SS has recently been shown to be highly relevant for the ability of *P. aeruginosa* strains to dominate in multi-species biofilms [17]. Notably, all three VgrG proteins that are co-regulated with this T6SS (VgrG1a-c) were upregulated, including VGR1c (MPAO1_11985; PA2685; 2.53 log2 FC). In contrast, none of the seven other members of the VgrG family (total of 10) [15] was expressed (see Supplementary **Data 1**).

Our pilot study proteomics dataset covered about 33% of the annotated MPAO1 proteins. This coverage was below that of the extensive proteomics dataset that had allowed to uncover expression evidence for all *Bartonella henseale* Type IV secretion system (T4SS) members [18]. However, that coverage was only be achieved by employing several elaborate fractionation and enrichment strategies, which was beyond the scope of this pilot study. Several of the structural members of the H1-T6SS include shorter proteins and membrane proteins, both of which are more difficult to detect by shotgun proteomics. Furthermore several of the secreted substrates of this H1-T6SS including Tse1 (PA1844, MPAO1_16450; 465 aa), Tse2 (PA2702, MPAO1_11900; 477 aa) and Tse3 (PA3484, MPAO1_07690; 1227 aa) are under tight and selective regulation [19] and were not detected in our study.

Supplementary Table 6. Summary table of annotation clusters created for the MPAO1 & PAO1 iPtgxDBs.

Supplementary Table 6A - MPAO1

Annotation source (tag in identifier)	Anno-tations	Clusters*	New clusters	New reductions	New extensions	Total clusters	Total ids
RefSeq (refseq)	5,851	5,851	5,851	0	0	5,851	5,851
Prodigal (prod)	5,691	5,691	55	309	150	5,906	6,365
ChemGenome (chemg)	4,616	4,616	1,703	81	1,745	7,609	9,894
In silico ORF (orf)	155,710	70,655	63,065	78	76,881	70,674	149,918

* See the original paper for a detailed description of the annotation clusters [20] or also the website https://iptgxdb.expasy.org/creating_iptgxdbs/ for more information.
The final iPtgxDB contained 146,826 entries, as sequences <6 aa (2,778), i.e., not identifiable with shotgun proteomics, annotated as pseudogenes in all annotations (47), and indistinguishable internal start sites (267) were not considered.

Supplementary Table 6B - PAO1

Annotation source (tag in identifier)	Anno-tations	Clusters*	New clusters	New reductions	New extensions	Total clusters	Total ids
RefSeq 2019 (refseq)	5,592	5,591	5,591	0	1	5,591	5,592
RefSeq 2017 (RS17)	5,577	5,577	45	31	8	5,636	5,676
Genoscope (geno)	5,835	5,835	202	11	3	5,838	5,892
Prodigal (prod)	5,681	5,681	27	294	160	5,865	6,373
ChemGenome (chemg)	4,738	4,738	1,795	66	1,719	7,660	9,953
In silico ORF (orf)	155,518	70,445	62,788	121	76,199	70,448	149,061

The final iPtgxDB contained 145,978 entries, as sequences <6 aa (2,788), i.e., not identifiable with shotgun proteomics, annotated as pseudogenes in all annotations (18), and indistinguishable internal start sites (277) were not considered.

Supplementary Data 5. Design of the microfluidic chamber as a CAD file.

See separate DWG file.

References

1. Olivas, A.D., et al., *Intestinal tissues induce an SNP mutation in Pseudomonas aeruginosa that enhances its virulence: possible role in anastomotic leak.* PLoS One, 2012. **7**(8): p. e44326.
2. Chandler, C.E., et al., *Genomic and Phenotypic Diversity among Ten Laboratory Isolates of Pseudomonas aeruginosa PAO1.* J Bacteriol, 2019. **201**(5): p. pii: e00595-18.
3. Lee, S.A., et al., *General and condition-specific essential functions of Pseudomonas aeruginosa.* Proc Natl Acad Sci U S A, 2015. **112**(16): p. 5189-5194.
4. Turner, K.H., et al., *Essential genome of Pseudomonas aeruginosa in cystic fibrosis sputum.* Proc Natl Acad Sci U S A, 2015. **112**(13): p. 4110-4115.
5. Stover, C.K., et al., *Complete genome sequence of Pseudomonas aeruginosa PAO1, an opportunistic pathogen.* Nature, 2000. **406**(6799): p. 959-964.
6. Klockgether, J., et al., *Genome diversity of Pseudomonas aeruginosa PAO1 laboratory strains.* J Bacteriol, 2010. **192**(4): p. 1113-1121.
7. Page, A.J., et al., *Roary: rapid large-scale prokaryote pan genome analysis.* Bioinformatics, 2015. **31**(22): p. 3691-3693.
8. Duong, F., et al., *The AprX protein of Pseudomonas aeruginosa: a new substrate for the Apr type I secretion system.* Gene, 2001. **262**(1-2): p. 147-53.
9. Borlee, B.R., et al., *Pseudomonas aeruginosa uses a cyclic-di-GMP-regulated adhesin to reinforce the biofilm extracellular matrix.* Mol Microbiol, 2010. **75**(4): p. 827-42.
10. Mah, T.F., *Establishing the minimal bactericidal concentration of an antimicrobial agent for planktonic cells (MBC-P) and biofilm cells (MBC-B).* J Vis Exp, 2014(83): p. e50854.
11. Lampaki, D., A. Diepold, and T. Glatter, *A Serial Sample Processing Strategy with Improved Performance for in-Depth Quantitative Analysis of Type III Secretion Events in Pseudomonas aeruginosa.* J Proteome Res, 2020. **19**(1): p. 543-553.
12. Herbst, F.A., et al., *Major proteomic changes associated with amyloid-induced biofilm formation in Pseudomonas aeruginosa PAO1.* J Proteome Res, 2015. **14**(1): p. 72-81.
13. Toyofuku, M., et al., *Identification of proteins associated with the Pseudomonas aeruginosa biofilm extracellular matrix.* J Proteome Res, 2012. **11**(10): p. 4906-15.
14. Kumari, H., et al., *LTQ-XL mass spectrometry proteome analysis expands the Pseudomonas aeruginosa AmpR regulon to include cyclic di-GMP phosphodiesterases and phosphoproteins, and identifies novel open reading frames.* J Proteomics, 2014. **96**: p. 328-342.
15. Hachani, A., et al., *Type VI secretion system in Pseudomonas aeruginosa: secretion and multimerization of VgrG proteins.* J Biol Chem, 2011. **286**(14): p. 12317-12327.
16. Allsopp, L.P., et al., *RsmA and AmrZ orchestrate the assembly of all three type VI secretion systems in Pseudomonas aeruginosa.* Proc Natl Acad Sci U S A, 2017. **114**(29): p. 7707-7712.
17. Cheng, Y., et al., *Population dynamics and transcriptomic responses of Pseudomonas aeruginosa in a complex laboratory microbial community.* NPJ Biofilms Microbiomes, 2019. **5**: p. 1.
18. Omasits, U., et al., *Directed shotgun proteomics guided by saturated RNA-seq identifies a complete expressed prokaryotic proteome.* Genome Res, 2013. **23**(11): p. 1916-1927.
19. Hood, R.D., et al., *A type VI secretion system of Pseudomonas aeruginosa targets a toxin to bacteria.* Cell Host Microbe, 2010. **7**(1): p. 25-37.
20. Omasits, U., et al., *An integrative strategy to identify the entire protein coding potential of prokaryotic genomes by proteogenomics.* Genome Res, 2017. **27**(12): p. 2083-2095.
