# Supplementary material for: An integrated model system to gain mechanistic insights into biofilm-associated antimicrobial resistance in Pseudomonas aeruginosa MPAO1
Source: NPJ Biofilms Microbiomes. 2020 Oct 30;6:46. doi: 10.1038/s41522-020-00154-8 (PMC7603352; doi:10.1038/s41522-020-00154-8)
Supplement: Supplementary file 7 — Reporting Summary Checklist [file 41522_2020_154_MOESM7_ESM.pdf]

nature research

Corresponding author(s): Adithi R. Varadarajan, Christian H. Ahrens

Last updated by author(s): Sep 21, 2020

Reporting Summary

Nature Research wishes to improve the reproducibility of the work that we publish. This form provides structure for consistency and transparency in reporting. For further information on Nature Research policies, see our Editorial Policies and the Editorial Policy Checklist.

Statistics

For all statistical analyses, confirm that the following items are present in the figure legend, table legend, main text, or Methods section.

n/a	Confirmed	
☐	☒	The exact sample size (*n*) for each experimental group/condition, given as a discrete number and unit of measurement
☐	☒	A statement on whether measurements were taken from distinct samples or whether the same sample was measured repeatedly
☐	☒	The statistical test(s) used AND whether they are one- or two-sided<br>*Only common tests should be described solely by name; describe more complex techniques in the Methods section.*
☒	☐	A description of all covariates tested
☐	☒	A description of any assumptions or corrections, such as tests of normality and adjustment for multiple comparisons
☐	☒	A full description of the statistical parameters including central tendency (e.g. means) or other basic estimates (e.g. regression coefficient) AND variation (e.g. standard deviation) or associated estimates of uncertainty (e.g. confidence intervals)
☐	☒	For null hypothesis testing, the test statistic (e.g. *F*, *t*, *r*) with confidence intervals, effect sizes, degrees of freedom and *P* value noted<br>*Give P values as exact values whenever suitable.*
☒	☐	For Bayesian analysis, information on the choice of priors and Markov chain Monte Carlo settings
☒	☐	For hierarchical and complex designs, identification of the appropriate level for tests and full reporting of outcomes
☒	☐	Estimates of effect sizes (e.g. Cohen's *d*, Pearson's *r*), indicating how they were calculated

Our web collection on statistics for biologists contains articles on many of the points above.

Software and code

Policy information about availability of computer code

Data collection	No custom software code was used for data collection in this study. All data used were either generated by the authors (details are provided in the methods section of the manuscript) or downloaded. Raw sequencing data from the PacBio RSII and Illumina MiSeq platforms were collected and further processed with open source software as described. Shotgun proteomics data were generated on a . Thermo Scientific Orbitrap Instrument. To identify essential P. aeruginosa MPAO1 genes (previously missed due to genomic blind spots and mapping data to the PAO1 reference strain), we downloaded MPAO1 Tn-Seq data from NCBI's SRA (SRP052838) using NCBI's FTP server.
Data analysis	De novo genome assembly was carried out using open software tools (the respective versions are listed in the Methods). Tn-seq data was analyzed using the scripts released by the authors of a previous study (Lee et al, PNAS 2015; see Ref#24), following their data analysis steps and cut-offs to call a gene essential. Confocal image analysis to assess the interlaboratory reproducibility of the microfluidic flow chamber was performed using COMSTAT 2.1 (IMAGE J), and statistical analyses performed using GraphPad Prism version 8. The data analysis from the screening of mutant strains for biofilm formation or biofilm-mediated resistance to colistin was performed with a Student's t-test. Proteomics data were searched using Proteome Discoverer Software and MS-GF+. Differential protein abundance was analyzed with DESeq2 (multiple-testing corrected) as described in the methods.

For manuscripts utilizing custom algorithms or software that are central to the research but not yet described in published literature, software must be made available to editors and reviewers. We strongly encourage code deposition in a community repository (e.g. GitHub). See the Nature Research guidelines for submitting code & software for further information.

Data

Policy information about availability of data

All manuscripts must include a data availability statement. This statement should provide the following information, where applicable:

- Accession codes, unique identifiers, or web links for publicly available datasets
- A list of figures that have associated raw data
- A description of any restrictions on data availability

The MPAO1 genome sequence is available at NCBI Genbank (acc# CP027857; Bioproject: PRJNA438597, Biosample: SAMN08722738). Read data are available under SRR10153205 (Illumina) and SRR10153206 (PacBio). Proteomics data are available from PRIDE (acc# PXD017122) upon acceptance of the manuscript. The iPtgxDBs for P. aeruginosa MPAO1 and PAO1 are available from https://iptgxdb.expasy.org, both as a searchable protein database (FASTA format) and a GFF file, which can be loaded in a genome viewer and overlaid with experimental evidence. Biofilm growth data from the microfluidic chamber will be made available at https://doi.org/10.21253/DMU.c.4851483 upon acceptance of the manuscript. The screening for biofilm formation and colistin resistance datasets generated and/or analyzed during the current study are available from the corresponding author on reasonable request. To support technology dissemination, the polyurethane master molds of the microfluidic chambers are available upon request from the UoS/NBIC; a CAD file can be found as Supplementary File 11. Code availability: all analyses presented rely on open source software or published code that are referenced.

Field-specific reporting

Please select the one below that is the best fit for your research. If you are not sure, read the appropriate sections before making your selection.

☒ Life sciences ☐ Behavioural & social sciences ☐ Ecological, evolutionary & environmental sciences

For a reference copy of the document with all sections, see nature.com/documents/nr-reporting-summary-flat.pdf

Life sciences study design

All studies must disclose on these points even when the disclosure is negative.

Sample size	No sample size calculation was performed for this proof of principle study. Close to 100 clones of a publicly available transposon insertion library were tested and compared to positive and negative control strains (WT and selected mutant strains), allowing us to showcase the utility of the screening experiments to identify genes with an effect on biofilm formation and biofilm-associated resistance to colistin. Selected candidates were tested in a larger biofilm flow chamber and provided reproducible results. The utility and reproducibility of the biofilm flow chamber was furthermore confirmed in an inter-laboratory trial across three consortium laboratories.
Data exclusions	Biofilm formation was tested with WT, selected positive and negative controls and mutant strains contained on one microtiter plate of the transposon mutant collection (overall WT and 96 mutant strains). A few data points were not included as they showed highest variability in biofilm formation or biofilm resistance (three data points each). To be consistent, the 3 mutants removed due to high variation of biofilm formation were also removed from the assay assessing their biofilm-associated resistance (and vice-versa). So a total of 6 mutants were removed, (about 6% total).
Replication	Experiments were carried out with at least two biological replicates (for the screening) or more. For the confirmation of biofilm formation of interesting strains serving as reference values (WT, PA3552, PA4726), 11 biological repeats were done, for the other strains 4 biological replicates (and 5 technical replicates each). The testing for planktonic or biofilm-associated resistance to colistin (MIC and MBIC) involved two biological replicates (and 2 technical replicates each), i.e. 4 samples.
Randomization	Analyses compared the behaviour of WT cells (P. aeruginosa MPAO1) and mutant strains. Except for a few positive and negative controls chosen based on prior literature knowledge, the strains were selected randomly from the transposon mutant library and analysed both for biofilm formation and for biofilm-associated resistance.
Blinding	No blinding as this was not relevant to our study (no patient samples; mere analysis of biological replicates of experiments providing standard deviations where appropriate).

Reporting for specific materials, systems and methods

We require information from authors about some types of materials, experimental systems and methods used in many studies. Here, indicate whether each material, system or method listed is relevant to your study. If you are not sure if a list item applies to your research, read the appropriate section before selecting a response.

Materials & experimental systems

n/a	Involved in the study
☒	☐ Antibodies
☒	☐ Eukaryotic cell lines
☒	☐ Palaeontology and archaeology
☒	☐ Animals and other organisms
☒	☐ Human research participants
☒	☐ Clinical data
☒	☐ Dual use research of concern

Methods

n/a	Involved in the study
☒	☐ ChIP-seq
☒	☐ Flow cytometry
☒	☐ MRI-based neuroimaging
